# Multifunctional nanomedicine targeting the 'seed-and-soil' of hair follicles via simultaneous alleviation of oxidative stress and activation of autophagy for androgenetic alopecia therapy

**DOI:** 10.1016/j.mtbio.2025.102145

**Published:** 2025-07-29

**Authors:** Yuanzheng Chen, Qubo Zhu, Yanbin Zhou, Wenhu Zhou, Yan Chen

**Affiliations:** a Xiangya School of Pharmaceutical Sciences, Central South University, Changsha, Hunan, 410013, China; b Quzhou Hospital Affiliated to Wenzhou Medical University (Quzhou People's Hospital), Quzhou, 324000, China; c Hunan Key Laboratory of The Research and Development of Novel Pharmaceutical Preparations, School of Pharmaceutical Science, Changsha Medical University, Changsha, 410219, China

**Keywords:** Androgenetic alopecia (AGA), Nanoparticles, Curcumin, Metal-organic frameworks, Microneedle, Reactive oxygen species (ROS) scavenging

## Abstract

Androgenetic alopecia (AGA) is a prevalent form of hair loss, which significantly affects both aesthetics and quality of life. Hair regeneration in AGA requires the transition of hair follicles from the telogen phase to the anagen phase, alongside a healthy microenvironment, analogous to seed germination, where breaking dormancy and providing fertile soil are essential. In this context, we propose that targeting the "seed-and-soil" model by activating autophagy in hair follicles (seeds) and alleviating oxidative stress in the hair follicle microenvironment (soil) could be an effective strategy for AGA treatment. Through network pharmacology and cell-based experiments, we identified curcumin (Cur) as a potential agent capable of activating autophagy and alleviating oxidative stress—both critical processes for hair follicle regeneration. To facilitate biomedical application, we developed a novel nanoparticle formulation, TFC, achieved by self-assembling Cur and tannic acid with Fe^3+^ via metal coordination. TFC nanoparticles demonstrated excellent colloidal stability, high Cur loading capacity (52 %), and potent antioxidant properties. In vitro studies showed that TFC effectively scavenged reactive oxygen species (ROS) and activated autophagy in human dermal papilla cells, offering significant protection against oxidative stress. In an AGA mouse model, TFC delivered via microneedles accelerated hair growth, promoted hair follicle proliferation, and enhanced angiogenesis, with superior efficacy compared to minoxidil and minimal side effects. This study suggests that Cur-loaded TFC, by targeting oxidative stress and autophagy in hair follicle cells, represents a promising novel approach for AGA treatment.

## Graphical abstract

(A) We developed a novel Cur-loaded TFC nanoparticle formulation using a metal coordination self-assembly strategy, achieved through a simple one-step mixing process. (B) The TFC nanoparticles exhibited enzyme-like activity and broad-spectrum free radical scavenging properties, alleviating the hypoxic microenvironment associated with AGA. (C) Delivered via microneedles, the TFC nanoparticles effectively reached deeper hair follicles, improving the follicular microenvironment (soil) and activating autophagy in hair follicle cells (seeds), offering a promising therapeutic approach for AGA treatment.

**Figure:**
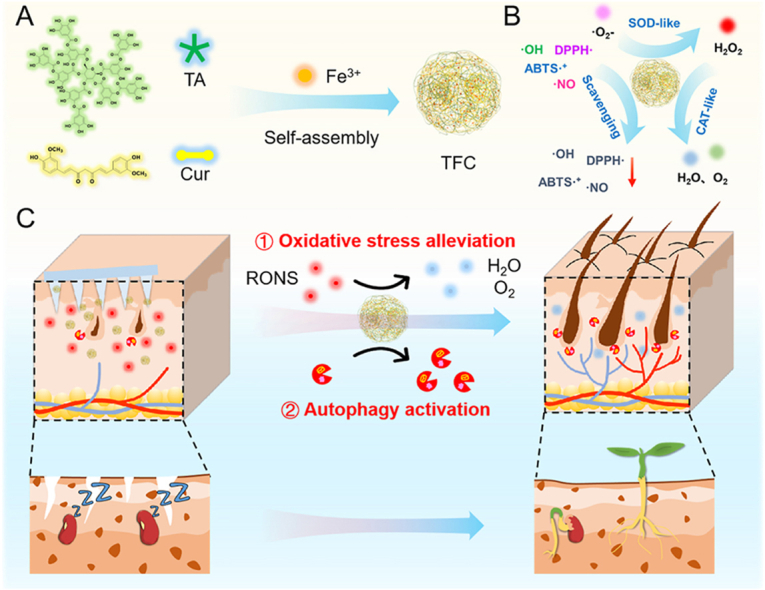

## 1 Introduction

Hair follicles are the primary structures responsible for hair growth, exhibiting robust self-renewal capabilities and undergoing periodic growth cycles [1,2]. The normal hair growth cycle comprises three distinct phases: the anagen (growth) phase, the catagen (degeneration) phase, and the telogen (resting) phase, with approximately 85 % of hair in the anagen phase anytime [3,4]. Disruptions in this normal cycle can lead to hair loss. Androgenetic alopecia (AGA) is one of the most prevalent forms of hair loss, characterized by progressive hair thinning and loss on the forehead and crown, affecting over 50 % of individuals above the age of 50 [5]. AGA not only impairs aesthetics but also has a significant impact on mental health, contributing to a reduction in quality of life [6,7].

AGA results from a combination of factors, including androgenic hormones, microvascular insufficiency, and oxidative stress [8,9]. Testosterone, once converted into dihydrotestosterone (DHT) by 5α-reductase within the hair follicles, binds strongly to androgen receptors, thereby activating downstream signaling pathways that shorten the anagen phase and prolong the telogen phase [10,11]. While oral finasteride, a 5α-reductase inhibitor, can mitigate the effects of androgens on hair follicles, prolonged use of the drug is associated with adverse sexual side effects, including decreased libido and erectile dysfunction in male patients [12]. Moreover, the microenvironment of hair follicles plays a crucial role in hair growth, and dysregulation of this environment is another contributing factor to hair loss [13]. Oxidative stress induces aging and apoptosis of hair follicle cells, while inadequate microvascular formation limits nutrient supply to the hair follicles, impeding their transition from the telogen phase to the anagen phase and slowing hair growth [14,15]. Topical minoxidil, a common treatment for AGA, exerts its therapeutic effects by promoting vasodilation, thereby enhancing blood flow to hair follicles and stimulating the transition from the telogen to the anagen phase [16]. However, the local application of minoxidil often struggles to penetrate deep into the skin, and prolonged use can result in adverse reactions such as allergic dermatitis [17]. Therefore, the development of highly effective and low-toxicity therapies that target the underlying pathophysiology of AGA is of paramount clinical importance.

In terms of AGA, hair follicle dormancy can be compared to seed dormancy, with the follicular microenvironment serving as the "soil" for hair regeneration. Successful hair regeneration in AGA requires the transition of hair follicles from a dormant (telogen) phase to an active (anagen) phase, akin to seed germination, which requires breaking dormancy and access to fertile soil. Increasingly, studies show a strong relationship between androgenetic alopecia and autophagy [[18], [19], [20], [21]]. Recent studies have shown that early degeneration of miniaturized hair follicles in AGA leads to increased apoptosis and severe impairment of endogenous autophagy [22]. Autophagy activators have been shown to promote the transition of hair follicles from the telogen to the anagen phase, stimulating hair growth [23]. Conversely, autophagy inhibitors induce apoptosis in hair follicle cells, leading to premature follicle degeneration [22]. Autophagy is an evolutionarily conserved intracellular process that maintains cellular homeostasis and adapts to adverse conditions, such as nutrient deprivation, by removing damaged or aging cellular components [24,25]. Thus, activating autophagy in hair follicles may be a promising strategy to stimulate the transition of hair follicles from the telogen phase to the anagen phase in AGA. Moreover, oxidative stress has been implicated in creating a hostile environment for hair follicle growth in AGA [26]. Excessive oxidative stress leads to aging and apoptosis of hair follicle cells, inhibits keratinocyte proliferation, and disrupts the differentiation of hair follicle stem cells by inducing the secretion of inflammatory cytokines [27]. Therefore, mitigating oxidative stress is a critical approach for improving the follicular microenvironment in AGA. Based on this "seed-soil" model, we propose that activating autophagy in hair follicles (the seeds) and alleviating oxidative stress in the follicular microenvironment (the soil) may offer a promising therapeutic strategy for treating AGA. However, to date, no studies have explored this combined approach.

Curcumin (Cur), a natural polyphenolic compound derived from turmeric, possesses potent anti-inflammatory and antioxidant properties [28]. Recent studies have demonstrated that Cur can activate autophagy, providing therapeutic benefits in conditions such as atherosclerosis, acute renal injury, and osteoarthritis [[29], [30], [31]]. Building on these findings, this work first investigated the potential mechanisms by which Cur alleviates oxidative stress and activates autophagy in terms of AGA. Through a combination of network pharmacology and cell-based experiments, we identify Cur's dual pharmacological effects: scavenging free radicals to mitigate oxidative stress and inhibiting intracellular oxidative damage via the suppression of c-Jun phosphorylation. Additionally, Cur inhibits the phosphorylation of Akt and mTOR, which in turn promotes the expression of LC3B and activates the autophagy pathway. Thus, Cur's ability to both reduce oxidative stress and stimulate autophagic activity positions it as a promising therapeutic candidate for AGA.

**Fig. 1:**
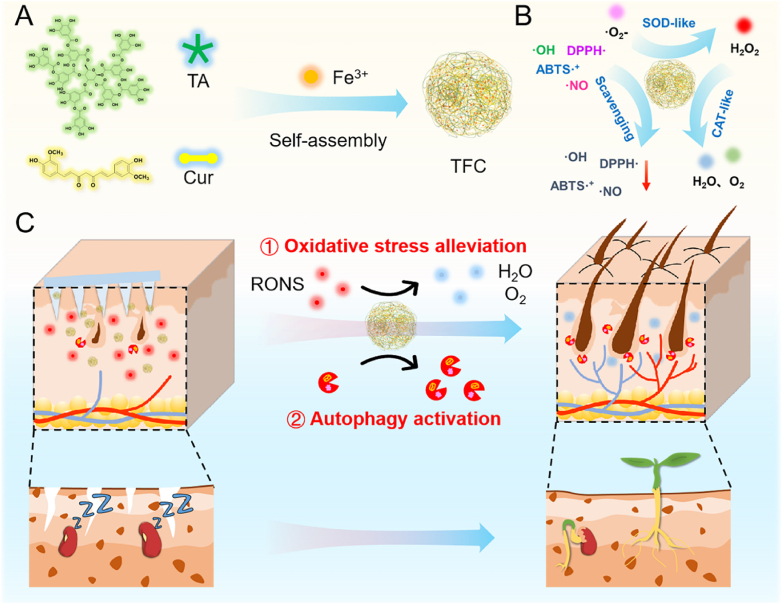
(A) Schematic diagram of TFC construction: Curcumin (Cur) and tannic acid (TA) coordinate with Fe^3+^ to form Cur-loaded TFC nanoparticles through a simple one-step self-assembly process. (B) Schematic diagram of the reactive oxygen and nitrogen species (RONS) clearance ability and enzyme-like activity of TFC. (C) Mechanism of TFC treatment for AGA: TFC nanoparticles, delivered transdermally via microneedles, improved the follicular microenvironment (soil) via RONS scavenging and activated autophagy in hair follicle cells (seeds), promoting hair regeneration in the AGA mice model.

Despite its promising pharmacological effects, Cur's clinical applications are limited by its poor water solubility and low stability [32,33]. To overcome these challenges, a variety of nanocarrier systems have been developed for Cur delivery, including micelles, liposomes, and polymer nanoparticles [34,35]. In this study, we developed a novel nanocarrier formulation using a metal coordination self-assembly strategy to enhance the solubility and stability of Cur. Specifically, we used tannic acid (TA) and Cur as co-ligands to coordinate with Fe^3+^ to form Cur-loaded TFC nanoparticles through a simple one-step mixing process (Fig. 1A). The TFC nanoparticles exhibited uniform structure, high colloidal stability, 100 % encapsulation efficiency, and a drug loading capacity of 52 %. The integration of TA and Cur endowed TFC nanoparticles with broad-spectrum free radical scavenging activity, while the Fe^2+^/Fe^3+^ redox cycle within the structure imparts superoxide dismutase and catalase-like activities, which help to scavenge superoxide anions and decompose hydrogen peroxide, thus alleviating the hypoxic microenvironment at the site of AGA (Fig. 1B). The TFC nanoparticles also released Cur to activate autophagy, providing cellular protection. Following transdermal delivery via microneedles, TFC nanoparticles were effectively transported to the deeper hair follicles, where they promote AGA hair regeneration by improving the follicular microenvironment (fertilize the soil) and activating autophagy (rouse from dormancy) in hair follicle cells (Fig. 1C). In a mouse AGA model, TFC demonstrated superior therapeutic efficacy compared to minoxidil, with excellent biosafety. This study proposed a novel approach to AGA treatment based on the "seed-soil" model, providing evidence for Cur's potential as a therapeutic agent for AGA and highlighting the promise of TFC as a nanocarrier for Cur delivery.

## 2 Materials and methods

### 2.1 Materials

Curcumin (L830W30) was purchased from J&K Scientific (Beijing, China). Tannic acid (C14951039), 2,2′-Azino-bis (3-ethylbenzothiazoline-6-sulfonic acid) diammonium salt (ABTS) and dihydroethidium (DHE) were purchased from Macklin Co., Ltd. (Shanghai, China). FeCl_3_·6H_2_O (20230612) and PVP K30 (20190828) were purchased from Sinopharm Chemical Reagent Co., Ltd (Shanghai, China). 1,1-diphenyl-2-picrylhydrazyl (DPPH) was from Shanghai Biological Technology Development Co., Ltd. (Shanghai, China). Sodium hyaluronate (N25IS232980) was purchased from Shanghai yuanye Bio-Technology Co., Ltd (Shanghai, China). Testosterone (F2206484) was purchased from Shanghai Aladdin Biochemical Technology Co., Ltd (Shanghai, China). 2′,7′-Dichlorodihydrofluorescein diacetate (DCHF-DA) was obtained from Sigma-Aldrich (St. Louis, USA). 4-amino,5-aminomethyl-2′,7′-difluorescein diacetate (DAF-FM DA) was purchased from Beyotime (Shanghai, China). Hydroxyphenyl fluorescein (HPF) was obtained from Maokang Biotechnology Co., Ltd. (Shanghai, China). The anti-p-Akt antibody, anti-Akt antibody, anti-p-mTOR antibody, anti-mTOR antibody, anti-LC3B antibody, anti-p-c-Jun antibody were purchased from Hangzhou HUABIO Biotechnology Co., Ltd (China), and anti-c-Jun antibody was purchased from Cell Signaling Technology (USA). Anti-Ki67 antibody and anti-CD31 antibody were purchased from Abcam Trading Co., Ltd (Shanghai, China). Minoxidil gel was purchased from Bausch & Lomb (China). Human dermal papilla cells (DPCs) were purchased from Shanghai Boquaner Biotechnology Co., Ltd (Shanghai, China).

### 2.2 Network pharmacology analysis and molecular docking

Genes associated with androgenetic alopecia were screened as candidate targets using the PubChem database (https://pubchem.ncbi.nlm.nih.gov/pathway/) as well as the WikiPathways database. Candidate targets as well as curcumin were entered into stitch (http://stitch.embl.de/) for protein-protein interaction (PPI) network visualization. In pathway enrichment analysis, the latest GO annotations and KEGG pathway annotations of the genes were obtained from KEGG REST API (https://www.kegg.jp/kegg/rest/keggapi.html) and GO and KEGG enrichment analyses were performed, setting the minimum gene set to 5 and the maximum gene set to 5000, and p value < 0.05 and FDR<0.25 as the significance index to screen the significant items and visualize them.

Crystal structures of human-derived JUN (PDB ID: 1S9K) and mTOR (PDB ID: 4JSV) were obtained from the RCSB Protein Data Bank (https://www.rcsb.org/). The 3D structure of curcumin was obtained from PubChem (https://pubchem.ncbi.nlm.nih.gov). Protein and curcumin pre-processing using AutoDock Tools. Molecular docking was performed in Autodock vina and visualized using PyMOL.

### 2.3 Protein expression validation of oxidative stress, autophagy pathway

DPCs were seeded in 6-well plates at a density of 20 × 10^4^/well and cultured overnight. Cur (5–20 μM) and H_2_O_2_ (1000 μM) were incubated with the cells for 4 h and 8 h, respectively. Cells were collected and lysed by adding RIPA lysate (containing 1 % PMSF and 1 % phosphatase inhibitor) at 4 °C for 30 min, and the supernatant was centrifuged at 12,000 rpm for 10 min at 4 °C as the total protein sample. The protein expression of oxidative stress and autophagy pathway was analyzed by Western Blotting.

### 2.4 Free radical scavenging ability of Cur

For DPPH• scavenging ability, 20 μL of different concentrations of Cur were mixed with 180 μL of acetic acid-sodium acetate buffer (100 mM, pH 5.5) and 200 μL of DPPH• (0.3 mM), respectively, and reacted for 30 min avoiding light. The absorbance at 517 nm and the UV absorption peaks at 450–650 nm were measured by a microplate reader (Infinite M200PRO, TECAN) at 5-min intervals. For ABTS•^+^ scavenging ability, 7.4 mM ABTS was mixed with 2.6 mM ammonium persulfate at 1:1 (v: v) and placed at 4 °C overnight to obtain ABTS•^+^. 50 μL of each concentration of TFC was mixed with 200 μL of ABTS•^+^, respectively, and the reaction was carried out for 30 min, avoiding light. The absorbance at 734 nm and the UV absorption peaks at 500–900 nm were measured by microplate reader at 5-min intervals.

### 2.5 Synthesis and characterization of TFC

Firstly, curcumin was dissolved using different aqueous and organic solutions, centrifuged at 12,000 rpm for 10 min. Then, Cur concentration in supernatant was determined using UV–vis spectrum photometer (UV-2600, Shimadzu). For TFC synthesis, tannic acid (40 mg/mL), curcumin (5 mg/mL), and FeCl_3_·6H_2_O (10 mg/mL) were added to a 20 % acetone solution under vigorous stirring and immediately sonicated. The acetone was removed from the solution using a rotary evaporator (MVP 10 basic, IKA). The TFC was collected by centrifugation (4 °C, 16,000 rpm, 20 min) and washed twice to obtain. Curcumin-free tannic acid-iron nanoparticles (TF) were prepared according to previously reported methods [36].

The particle size distribution, polydispersity index (PDI) and zeta potential were measured by Malvern Zeta Sizer Nano series (Nano ZS, Malvern Instruments). Different concentrations of Cur and TFC solutions were prepared using ultrapure water and allowed to stand to assess their dispersion stability. To evaluate light stability, the UV–Vis absorption spectra of TFC aqueous solution and Cur ethanol solution were measured after 2 h of sunlight exposure, focusing on changes in the characteristic absorption peak at 426 nm. Additionally, to investigate the alkaline stability, TFC and Cur (dissolved in DMSO) were added to buffer solutions with pH 9 and pH 11, and their UV–Vis spectra were recorded at various time points to monitor alterations in the 426 nm absorption peak of curcumin. High performance liquid chromatography (HPLC) (1260 infinity Ⅱ, Agilent) was used to determine the encapsulation efficiency (EE) and loading efficiency (LE) of curcumin. Transmission electron microscopy (TEM) (Titan G2-F20, FEI) was used to characterize the morphology and elements mapping. Ultraviolet–visible (UV–vis) spectra were determined by UV–vis spectrum photometer. Infrared spectra were determined by infrared spectrometer (Spectrum Two, Perkin Elmer). X-ray diffractometer (XRD) (Advance D8 X, Bruker) and X-ray photoelectron spectrometer (XPS) (ESCALAB250Xi, Thermo Fisher) were used to characterize the crystalline and elemental compositions and valence states, respectively. The stability of TFC in ultrapure water, Tris (10 mM, pH 7.4) and DMEM (10 % FBS) was measured by monitoring the particle size after 72 h of incubation. TFC was added to Tris-HCl buffer (pH 7.4) containing 1 % mouse skin homogenate to simulate the enzymatic environment of the skin. At designated time points, samples were collected and replaced with an equal volume of fresh medium. The iron content was then quantified using inductively coupled plasma-optical emission spectrometry (ICP-OES, iCAP PRO X, Thermo Fisher).

### 2.6 Molecular dynamics simulation

Desmond software was used for enhanced sampling molecular dynamics simulations-Replica Exchange with Solute Tempering (REST). During the simulation, the OPLS2005 force field was used to parameterize the tannic acid molecule and curcumin molecule, and the water molecule was modeled using TIP3P. Subsequently, the sampling results were analyzed by cluster analysis. For the central conformation of each cluster, we extracted the main representative conformations for further quantitative optimization and analysis. The B3LYP generalization and the D3 empirical dispersion correction (with Becke-Johnson damping) were applied throughout the calculations to quantitatively optimize the dominant conformations. The def2-SVP basis set was used for both geometry and frequency calculations. The BSSE (basis set overlap error) method with the def2-TZVP basis set was employed to further accurately calculate the interaction energy between the tannic acid and curcumin molecular complexes. The interactions of the system were analyzed using IGMH (Independent Gradient Model based on HirshMnld splitting).

### 2.7 Characterization of in vitro RONS scavenging ability and enzyme-mimic activity of TFC

For DPPH• scavenging ability, 20 μL of different concentrations of TFC were mixed with 180 μL of acetic acid-sodium acetate buffer (100 mM, pH 5.5) and 200 μL of DPPH (0.3 mM), and the reaction was carried out in the dark for 30 min, the absorbance at 517 nm and the UV–vis absorption peaks at 450–650 nm were measured by microplate reader at 5 min intervals.

For ABTS•^+^ scavenging ability, 7.4 mM ABTS was mixed with 2.6 mM ammonium persulfate at 1:1 (v:v) and placed at 4 °C overnight to obtain ABTS•^+^, which was diluted and used. 50 μL of each concentration of TFC was mixed with 200 μL of ABTS•^+^, respectively, and the reaction was conducted for 30 min, avoiding light, and the absorbance at 734 nm and 500–900 nm UV–vis absorption peaks were measured by microplate reader at 5 min intervals.

For •OH scavenging ability, hydroxyl radicals were generated by using H_2_O_2_-Mn^2+^ Fenton-like system and 40 μL of MB (100 μg/mL) was added, followed by mixing with different concentrations of TFC, and then reacted for 30 min in a water bath at 37 °C. The absorbance at 662 nm was measured by microplate reader. In addition, each concentration of TFC was mixed with FeSO_4_, DMPO, and H_2_O_2_, and the reaction was carried out for 5 min and then analyzed by electron paramagnetic resonance spectrometry.

For •NO scavenging ability, the absorbance of the reaction solution at 540 nm was measured by using nitric oxide detection kit (Nanjing Jiancheng Bioengineering Institute, China) to analyze the scavenging ability of different concentrations of TFC on •NO.

For •O_2_- scavenging capacity, the absorbance at 550 nm of the reaction solution was measured using superoxide anion detection kit (Nanjing Jiancheng Bioengineering Institute, China) to analyze the scavenging capacity of different concentrations of TFC for •O_2_-. In addition, each concentration of TFC was mixed with xanthine solution, xanthine oxidase solution, and DMPO, and incubated for 10 min and then analyzed by electron paramagnetic resonance spectrometry.

For H_2_O_2_ scavenging ability, different concentrations of TFC were mixed with H_2_O_2_ (4 M) in equal volume and incubated in a 37 °C water bath for 30 min to observe the oxygen production, while dissolved oxygen meter was used to monitor the dissolved oxygen content in the solution from 0 to 10 min.

### 2.8 Cytotoxicity study and cellular uptake of TFC

DPCs were seeded in 96-well plates at a density of 1 × 10^4^/well and cultured overnight. The cells were incubated with different concentrations of TFC (calculated as curcumin) for 24 h. 100 μL of MTT solution was added to each well for 4 h. Subsequently, 100 μL of DMSO was added, and the relative cell viability was calculated by measuring the absorbance at 490 nm with microplate reader.

DPCs were seeded in confocal dishes at a density of 4 × 10^4^/well and cultured overnight. TFC was incubated with the cells for 2, 4 and 8 h. After 4 % paraformaldehyde fixation, Hoechst 33342 was added to stain the nuclei of the cells and the fluorescence was observed by confocal fluorescence microscopy. Similarly, cells were collected and the fluorescence intensity was analyzed by flow cytometry.

### 2.9 Intracellular RONS scavenging and MDA level assay

DPCs were seeded in 24-well plates at a density of 10 × 10^4^/well and cultured overnight. TA, Cur, TF and TFC were incubated with the cells separately for 12 h. The drugs were then removed and replaced with 500 μM H_2_O_2_ to continue incubation for 24 h. DCFH-DA (10 μM), DHE (5 μM), HPF (20 μM), and DAF-FM DA (5 μM) probes were added to the cells to incubate them for 30, 30, 45, and 30 min, respectively, and the fluorescence was observed under fluorescence microscope. Similarly, the cells were collected and the fluorescence levels were analyzed by flow cytometry. In addition, intracellular malondialdehyde levels were detected using a lipid oxidation (MDA) detection kit (Beyotime, China).

### 2.10 Cytoprotective effects of TFC and live/dead cell assay

DPCs were seeded in 96-well plates at a density of 5 × 10^3^/well and incubated overnight.TFC (0–40 μM) and H_2_O_2_ (0–800 μM) were incubated with the cells for 12 h and 24 h, respectively. 100 μL of MTT solution was added to each well for 4 h. Subsequently, 100 μL of DMSO was added, and the relative cell viability was calculated by determining absorbance at 490 nm with microplate reader.

DPCs were seeded in 96-well plates at a density of 5 × 10^3^/well and incubated overnight. 40 μM TFC and 500 μM H_2_O_2_ were incubated with the cells for 12 h and 24 h, respectively. Cells were assayed for live and dead by using Calcein/PI Cell Activity and Cytotoxicity Assay Kit (Beyotime, China). The fluorescence results were observed under fluorescence microscope (Calcein AM: Ex/Em = 494/517 nm; PI: Ex/Em = 535/617 nm).

### 2.11 Activation of cellular autophagy activity by TFC

DPCs were seeded in 6-well plates at a density of 20 × 10^4^/well and cultured overnight. Rapamycin, TA, Cur, TF, TFC were incubated with the cells separately for 12 h. The drugs were then removed and replaced with complete medium to continue incubation for 24 h, followed by cell collection. Autophagosomes were detected using autophagy detection kit (KeyGEN Bio TECH, China) and fluorescence was observed by fluorescence microscopy. Transmission electron microscopy was used to observe the cellular autophagic structure. In addition, Western blotting was repeated three times to analyze the LC3 protein expression.

### 2.12 Preparation and characterization of TFC MN

The TFC solution was added to the PDMS molds and kept in a vacuum for 10 min to fill the cavities, scraping off more than the solution for reuse and ensuring that each cavity was filled with the solution. Subsequently, a mixture of 20 % PVP K30 and 7.5 % sodium hyaluronate was added to the molds and placed in a desiccator to dry, and the removed TFC MN was stored in the desiccator. The morphology of the microneedles was characterized by camera and scanning electron microscopy. ICP-OES for determination the drug loading of TFC MN. The mechanical property for the microneedles were assessed using a biomechanical testing machine, where the upper stainless steel plate was moved downward at 0.1 mm/s until the stainless steel plate touched the microneedle to start recording the force-displacement curve. To assess the mechanical strength of the microneedles, the isolated dorsal mouse skin of C57/BL6 mice was fixed on the plate, the TFC MN was pressed onto the skin for 5 min and then removed, the insertion rate was calculated, and the skin was subjected to hematoxylin and eosin (H&E) sectioning to determine the insertion depth. To evaluate the in vivo dissolution behavior of TFC MN, the microneedles were applied to the depilated dorsal skin of mice for 5 min and then removed. The residual length of the microneedles was subsequently observed under a microscope.

### 2.13 Animal

Male C57/BL6 mice (6 weeks old) were purchased and housed in the Department of Laboratory Animal Science of Central South University with free access to food and water. Mice were anesthetized using an intraperitoneal injection of 0.3 % sodium pentobarbital and euthanized for tissue. All the animal experiments were approved by the Experimental Animal Ethics Committee of Central South University (Approval No. CSU-2023-0266).

### 2.14 Therapeutic efficacy of TFC MN in AGA mice

On day 0 of the experiment, a 2 × 2 cm area of the dorsal skin of 7-week-old C57/BL6 mice was depilated with a shaver and depilation cream, and randomly divided into normal group, AGA group, minoxidil group, blank microneedle group (Blank MN), curcumin microneedle group (Cur MN) and TFC microneedle group (TFC MN). Beginning on day 1 of the experiment, all groups except the normal group applied 0.2 % testosterone solution (prepared in 50 % ethanol) at a dose of 0.1 mL/cm^2^ to the depilated area for 28 days. The normal group did not receive any treatment. The minoxidil group applied 50 μL of 2.2 % minoxidil gel to the depilated area on the dorsal side of each mouse until day 13 after depilation. For the Blank MN, Cur MN and TFC MN groups, mice were given blank microneedle patches, curcumin microneedle patches and TFC microneedle patches every 3 days after depilation until the 13th day after depilation. Pictures were taken every 7 days to record hair growth and the number of days it took for the skin to begin to darken. On day 28 after depilation, the length of neonatal hair in mice was measured, and the coverage of neonatal hair in mice was measured using Image J software.

### 2.15 Histologic and immunofluorescent staining of the skin

On day 14 after depilation, the skin of the depilated dorsal region of mice in the normal group, AGA group, the Minoxidil group, the Blank MN group, the Cur MN group and the TFC MN group were immersed in 4 % paraformaldehyde and liquid nitrogen, respectively, and pathologic sections were made for observation. In addition, the skin sections of each group, DHE probe, primary antibody (anti-Ki67, anti-CD31, anti-LC3B, anti-p-c-Jun, anti-p-Akt, anti-p-mTOR) were added and secondary antibody was added after overnight incubation at 4 °C. Cell nuclei were stained using DAPI and fluorescence was observed by fluorescence microscopy.

### 2.16 In vivo safety assessment

Body weight was recorded every 7 days since the mice were depilated. Mice were executed on day 28 after depilation, and the heart, liver, spleen, lungs and kidneys were taken and placed in 4 % paraformaldehyde for overnight fixation, H&E sections were performed and observed. In addition, skin, heart, liver, spleen, lung and kidney tissues were taken separately, added with aqua regia, and placed in a 60 °C water bath to heat the tissues overnight for ablation. The digested solution was centrifuged at 1000 g for 10 min, and the supernatant was diluted and analyzed for iron content in the tissues by ICP-OES.

### 2.17 Statistical analysis

All results were presented as mean ± standard deviation. Student's t-test and one-way ANOVA test were performed using GraphPad Prism 8.0 software. *p* < 0.05 was considered statistically significant (∗*p* < 0.05, ∗∗*p* < 0.01, ∗∗∗*p* < 0.001, ∗∗∗∗*p* < 0.0001).

## 3 Results and discussion

### 3.1 Exploration of the mechanism of Cur in regulating oxidative stress and cellular autophagy

**Fig. 2:**
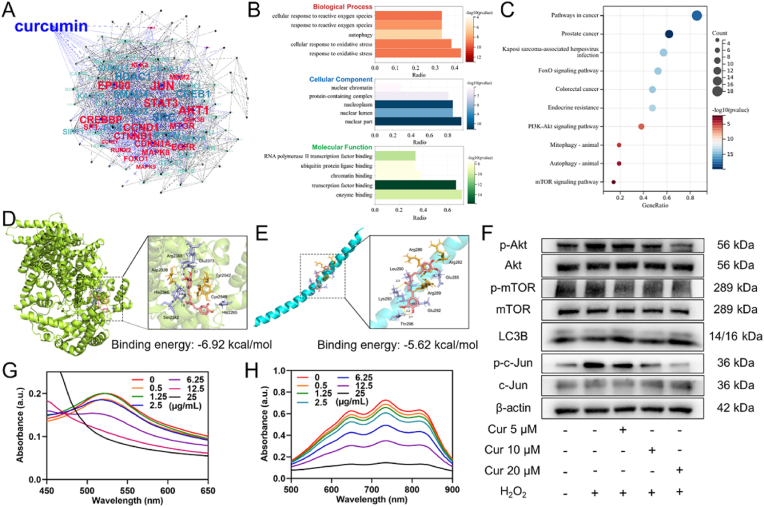
Screening, molecular docking, and validation of network pharmacology targets of Cur for alleviating oxidative stress and regulating autophagy. (A) Identification of the protein-protein interaction (PPI) network of Cur targets involved in alleviating oxidative stress and activating autophagy. (B) Gene Ontology (GO) functional enrichment analysis of Cur targets related to oxidative stress regulation and autophagy activation. (C) Kyoto Encyclopedia of Genes and Genomes (KEGG) pathway enrichment analysis of Cur targets involved in oxidative stress and autophagy. (D) Molecular docking between Cur and mTOR. (E) Molecular docking between Cur and c-Jun. (F) Western blot analysis to validate the regulatory effects of Cur on key oxidative stress and autophagy-related proteins. (G) UV spectral characterization of DPPH• scavenging activity by Cur at various concentrations. (H) UV spectral characterization of ABTS•^+^ radical clearance by Cur at different concentrations.

In this study, we first identified potential targets of Cur for treating AGA using network pharmacology, focusing on mechanisms related to the alleviation of oxidative stress and activation of autophagy. The results revealed that a total of 21 genes (highlighted in red) are directly associated with Cur (Fig. 2A). Among these, Jun is a key target in the oxidative stress pathway, and inhibition of its phosphorylation can effectively mitigate oxidative stress [37]. Akt1 and mTOR are crucial components in the autophagy pathway, with Akt1 acting upstream of mTOR. Inhibition of Akt1 and mTOR phosphorylation can stimulate autophagy and enhance the expression of downstream autophagy marker LC3B [38].

We performed Gene Ontology (GO) enrichment analysis and Kyoto Encyclopedia of Genes and Genomes (KEGG) pathway enrichment analysis on the 21 genes directly regulated by Cur. GO enrichment analysis revealed that biological processes (BP) include cellular response to reactive oxygen species, response to reactive oxygen species, autophagy, cellular response to oxidative stress and response to oxidative stress, cellular components (CC) including nuclear chromatin, protein-containing complex, nucleoplasm, nuclear lumen, nuclear part, and molecular functions (MF) including RNA Polymerase Ⅱ transcription factor binding, ubiquitin protein ligase binding, chromatin binding, transcription factor binding, enzyme binding (Fig. 2B). KEGG pathway analysis showed that Cur predominantly affects pathways associated with pathways in cancer, prostate cancer, Kaposi sarcoma-associated herpesvirus infection, FoxO signaling pathway, colorectal cancer, and endocrine resistance, PI3K-Akt signaling pathway, Mitophagy-animal, Autophagy-animal, mTOR signaling pathway (Fig. 2C). Molecular docking studies were conducted to assess the binding affinity between Cur and the target proteins mTOR and Jun. The results indicated that Cur interacts with mTOR and Jun primarily through non-covalent interactions, such as hydrogen bonds and van der Waals forces, with binding energy of −6.92 kcal/mol and −5.62 kcal/mol, respectively. (Fig. 2D–E).

Subsequently, we validated these findings at the cellular level in DPCs through Western blotting (WB) analysis, confirming Cur's regulatory effect on the phosphorylation of c-Jun, Akt, and mTOR, as well as the expression of LC3B in DPCs. The results demonstrated that Cur effectively inhibits the phosphorylation of c-Jun in an oxidative stress environment. Additionally, Cur downregulates the phosphorylation of Akt and mTOR, leading to the activation of the autophagy pathway and upregulation of LC3B expression (Fig. 2F and S1).

Furthermore, we investigated Cur's free radical scavenging activity and found that it effectively scavenges DPPH• and ABTS•^+^ radicals in vitro, within a concentration range of 25 μg/mL (Fig. 2G–H and S2). These findings suggested that Cur's ability to modulate oxidative stress is attributed to its dual pharmacological and chemical activities. Overall, our results highlighted the multifaceted roles of Cur in alleviating oxidative stress and inducing cellular autophagy, suggesting its potential as a therapeutic agent for AGA treatment.

### 3.2 Synthesis and characterization of TFC

**Fig. 3:**
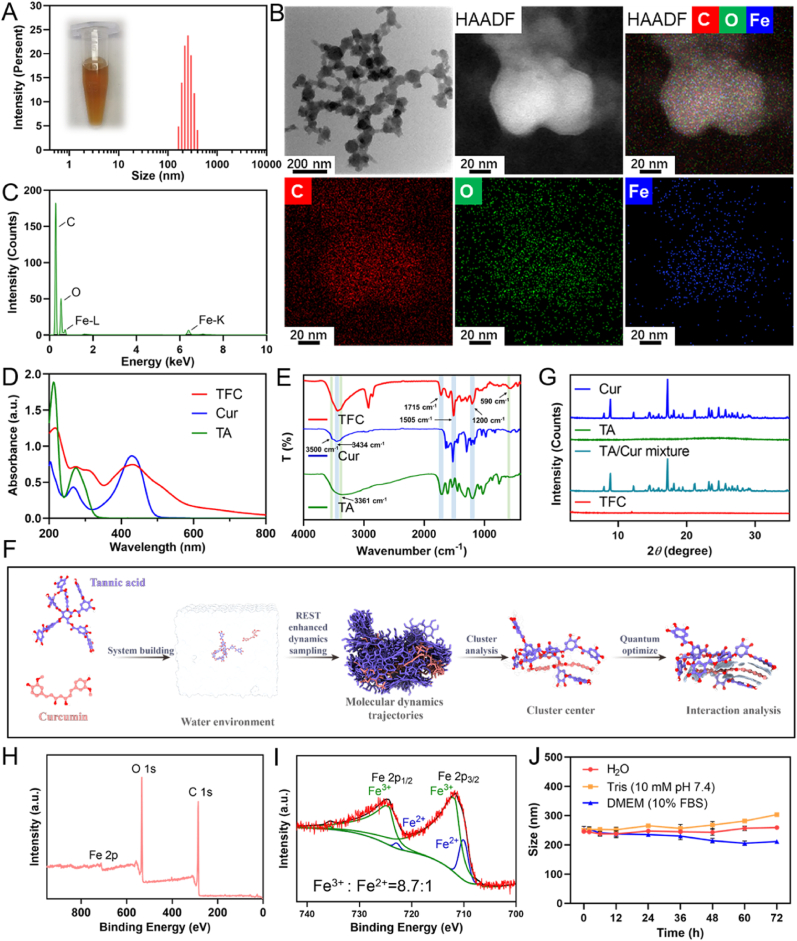
Synthesis and characterization of TFC. (A) Particle size distribution of TFC. Inset: Appearance of TFC solution. (B) Transmission electron microscopy (TEM) image of TFC. (C) Energy dispersive spectroscopy (EDS) spectrum of TFC. (D) UV–visible absorption spectra of TFC, Cur, and TA. (E) Infrared (IR) absorption spectra of TFC, Cur, and TA. (F) Molecular dynamics simulation of the self-assembly mechanism of TFC. (G) X-ray diffraction (XRD) patterns of TFC, Cur, TA, and TA/Cur mixtures. (H) X-ray photoelectron spectroscopy (XPS) spectrum of TFC. (I) XPS fine spectrum of Fe in TFC. (J) Particle size colloidal stability of TFC over 72 h in various solutions (n = 3).

After validation of its pharmacological activity, we measured the solubility of curcumin, the results showed that the solubility of curcumin in organic solutions was much better than that of aqueous solutions, indicating poor water solubility of curcumin (Fig. S3). Therefore, we developed a nanoformulation by loading Cur into TFC to address the solubility limitations of Cur. The construction of TFC is based on self-assembly through coordination between iron ions (Fe^3+^) and the Cur/TA complex (Fig. 1A). As illustrated in Fig. S4, the addition of iron ions to the TA/Cur mixed solution induces a color change from yellow to dark brown. Following centrifugation, the TFC nanoparticles are collected and redispersed in ultrapure water, resulting in an orange-yellow solution (Fig. 3A, inset). Dynamic light scattering (DLS) analysis revealed that the hydrated particle size of TFC is approximately 248 nm, with a polydispersity index (PDI) of 0.141, indicating a narrow particle size distribution and good dispersion (Fig. 3A). The surface potential of TFC was measured at −42 mV (Fig. S5), likely due to the presence of abundant carboxyl groups in the TA/Cur structure. The high negative charge on the nanoparticle surface promotes electrostatic repulsion between particles, thereby enhancing colloidal stability and minimizing aggregation [39]. TFC was uniformly dispersed in ultrapure water, whereas Cur precipitated, demonstrating that TFC significantly improved the aqueous solubility of Cur (Fig. S6A). Moreover, TFC enhanced the photostability of Cur under sunlight exposure and improved its stability in alkaline environments (Fig. S6B–D).

Transmission electron microscopy (TEM) imaging revealed that TFC nanoparticles have a spherical morphology, with a particle size of approximately 90 nm in the dry state (Fig. 3B). Elemental analysis of TFC using TEM and energy dispersive spectroscopy (EDS) confirmed the presence of three elements: carbon (C), oxygen (O), and iron (Fe) (Fig. 3B–C). The encapsulation efficiency (EE) of Cur was found to be 100 %, with a drug loading efficiency (LE) of 52 %, as determined by high-performance liquid chromatography (HPLC) (Fig. S7).

UV–visible spectroscopy analysis demonstrated that TFC exhibited a characteristic absorption peak of Cur at 426 nm and a peak for TA at 276 nm (Fig. 3D). Notably, due to the ligand-metal charge transfer (LMCT) effect, the absorption peak of Cur at 426 nm broadened, and a new absorption peak appeared between 490 and 550 nm, indicating coordination between Cur and Fe^3+^. TA exhibited an absorption peak at 213 nm, which showed a red shift in the TFC formulation. Additionally, new ligand-metal charge transfer absorption peaks were observed between 600 and 800 nm, indicating coordination between TA and Fe^3+^. A new absorption peak at 310 nm also appeared in TFC, which is likely associated with the formation of Fe-O coordination bonds in the TFC system. Infrared (IR) spectroscopy analysis of TFC revealed that the stretching vibration of the Cur phenolic hydroxyl group occurred at 3434 cm^−1^, the C=O stretching vibration of the TA ester group was at 1715 cm^−1^, the C=O and C=C vibrations of Cur were at 1505 cm^−1^, and the C-OH stretching vibration of TA occurred at 1200 cm^−1^, confirming the presence of both Cur and TA in the TFC formulation (Fig. 3E). Notably, the characteristic absorption peak of the phenolic hydroxyl group of Cur at 3500 cm^−1^ disappeared, the phenolic hydroxyl peak of TA shifted from 3361 cm^−1^ to 3434 cm^−1^, and a new Fe-O stretching vibration peak appeared at 590 cm^−1^, further confirming the coordination between TA/Cur and iron ions.

To gain insight into the self-assembly mechanism of TFC, molecular dynamics simulations were employed to analyze the interactions between TA, Cur, and Fe^3+^ (Fig. 3F). Cluster analysis of the TA and Cur complexes revealed that these molecules interlock tightly, primarily through intermolecular nesting and close packing (Fig. S8A). Upon the addition of Fe^3+^, the iron ions were positioned between the carbonyl and phenolic hydroxyl groups of the TA/Cur complex, forming stable interactions within the overall structure. Energy analysis indicated that the interaction between TA and Cur molecules is strong, facilitated by hydrogen bonding, π-π stacking, and hydrophobic interactions, with a binding energy of −27.119 kcal/mol (Fig. S8B). The inclusion of Fe^3+^ further enhanced this interaction, resulting in a binding energy of −43.853 kcal/mol. The Fe^3+^ ions form strong metal coordination bonds with the phenolic hydroxyl and carbonyl groups of TA and Cur, with a binding distance of approximately 2 Å and a binding energy of −485.721 kcal/mol, thus stabilizing the overall structure. These results suggested that TFC self-assembles primarily through coordination bonds, hydrogen bonding, π-π stacking, and hydrophobic interactions.

X-ray diffraction (XRD) analysis of TFC revealed that Cur exhibits a sharp crystalline peak in the 7–30° range, which disappears in TFC, indicating that Cur exists in an amorphous state within the nanoparticle formulation (Fig. 3G). X-ray photoelectron spectroscopy (XPS) analysis confirmed the presence of C, O, and Fe in TFC (Fig. 3H). The Fe 2p spectrum showed two prominent binding energy peaks at 711 eV (2p_3/2_) and 724 eV (2p_1/2_) (Fig. 3I), suggesting partial reduction of Fe^3+^ within the TFC structure. Quantitative analysis of Fe indicated that Fe^3+^ accounts for approximately 89.69 %, while Fe^2+^ comprises approximately 10.31 % of the iron present in TFC (Fig. 3I). The colloidal stability of TFC was further evaluated by incubating the nanoparticles in ultrapure water, Tris buffer (10 mM, pH 7.4), and DMEM (10 % FBS) for 72 h. No significant changes in particle size were observed, confirming the stability of TFC nanoparticles (Fig. 3J). The cumulative release of Fe in Tris-HCl buffer reached 36 % after 48 h (Fig. S9).

### 3.3 Characterization of in vitro RONS clearance ability and enzyme-mimic activity of TFC

**Fig. 4:**
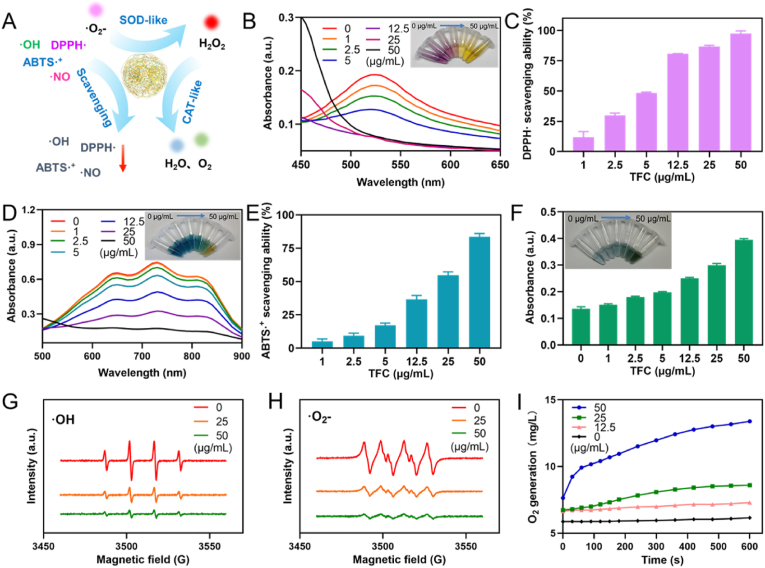
In vitro RONS clearance ability and enzyme-mimic activity characterization of TFC. (A) Schematic diagram illustrating the RONS clearance ability and enzyme-like activity of TFC. (B) UV spectroscopic characterization of DPPH• scavenging by TFC at different concentrations. Inset: Photographs of test tubes containing TFC at different concentrations reacting with DPPH• solution. (C) Quantitative analysis of DPPH• clearance rate at different TFC concentrations (n = 3). (D) UV spectroscopic characterization of ABTS•^+^ scavenging by TFC at different concentrations. Inset: Photographs of test tubes containing TFC at different concentrations reacting with ABTS•^+^ solution. (E) Quantitative analysis of ABTS•^+^ clearance rate at different TFC concentrations (n = 3). (F) UV absorbance of methylene blue (MB) used to characterize TFC's scavenging effect on •OH at different concentrations (n = 3). Inset: Photographs of test tubes containing TFC at different concentrations reacting with MB solution. (G) ESR plot showing TFC scavenging activity against •OH at different concentrations. (H) ESR plot showing TFC scavenging activity against •O_2_^−^ at different concentrations. (I) Dynamic curve of dissolved oxygen generation by TFC during H_2_O_2_ decomposition. (For interpretation of the references to color in this figure legend, the reader is referred to the Web version of this article.)

Cur and TA are both natural reducing agents, and the valence cycling between Fe^2+^ and Fe^3+^ in the TFC structure provides the nanoparticles with potential enzyme-mimic activity to scavenge reactive oxygen and nitrogen species (RONS) [40]. Specifically, the Fe^2+^ site mimics the activity of superoxide dismutase (SOD) by scavenging superoxide anions (•O_2_^−^) and hydroxyl radicals (•OH) through redox reactions, while the Fe^3+^ site mimics catalase by decomposing hydrogen peroxide (H_2_O_2_) into water and oxygen (Fig. 4A) [[41], [42], [43]]. To evaluate the RONS clearance ability and enzyme-mimic activity of TFC, we conducted a series of in vitro assays.

First, the antioxidant capacity of TFC was assessed using the DPPH• and ABTS•^+^ assays. Upon incubation with TFC at various concentrations, a gradual decrease in the characteristic absorption peak of DPPH• at 517 nm was observed, indicating the scavenging of DPPH• radicals (Fig. 4B). Quantitative analysis confirmed that TFC exhibited a concentration-dependent scavenging activity against DPPH•, with a clearance rate of 97.42 % at 50 μg/mL (Fig. 4C and S10). Similarly, TFC demonstrated effective scavenging of ABTS•^+^, as evidenced by the reduction in absorbance at 734 nm (Fig. 4D). At 50 μg/mL, the ABTS•^+^ clearance rate reached 83.51 % (Fig. 4E and S11).

To evaluate the ability of TFC to scavenge hydroxyl radicals (•OH), we employed methylene blue (MB) as an indicator. As the TFC concentration increased, the absorbance of MB at 662 nm gradually increased, indicating effective clearance of •OH radicals (Fig. 4F). Electron spin resonance (ESR) spectroscopy further confirmed that TFC effectively scavenged •OH, as the ESR signal intensity decreased with increasing TFC concentration (Fig. 4G). Additionally, TFC demonstrated good nitric oxide (·NO) scavenging ability, as evidenced by the decrease in absorbance at 540 nm after incubation with TFC at different concentrations (Fig. S12).

Next, we assessed the ability of TFC to simulate the activity of superoxide dismutase (SOD) by clearing superoxide anions (•O_2_^−^). TFC effectively scavenged •O_2_^−^, with a clearance rate of 71 % at a concentration of 50 μg/mL (Fig. 4H and S13). Hydrogen peroxide (H_2_O_2_) clearance, simulating catalase activity, was also evaluated. TFC demonstrated significant H_2_O_2_ decomposition activity, as indicated by the increased oxygen production upon H_2_O_2_ incubation (Fig. 4I and S14). The amount of dissolved oxygen generated increased with TFC concentration, suggesting that TFC not only decomposes H_2_O_2_ but also alleviates hypoxic conditions at the site of action by generating oxygen. The combined results from these assays confirmed that TFC exhibits broad-spectrum RONS scavenging abilities and mimics the enzymatic activities of SOD and catalase, making it a promising candidate for mitigating oxidative stress and hypoxia in therapeutic applications.

### 3.4 Intracellular free radical scavenging activity of TFC

After confirming the RONS clearance ability of TFC at the test tube level, we next investigated its cellular biological activity using dermal papilla cells (DPCs) as a model. DPCs are specialized fibroblasts that regulate the hair growth cycle, and their proliferation is essential for hair follicle development. Oxidative stress is known to cause aging and apoptosis in DPCs, which can disrupt hair growth [44]. Therefore, we evaluated the intracellular free radical scavenging activity of TFC in DPCs.

**Fig. 5:**
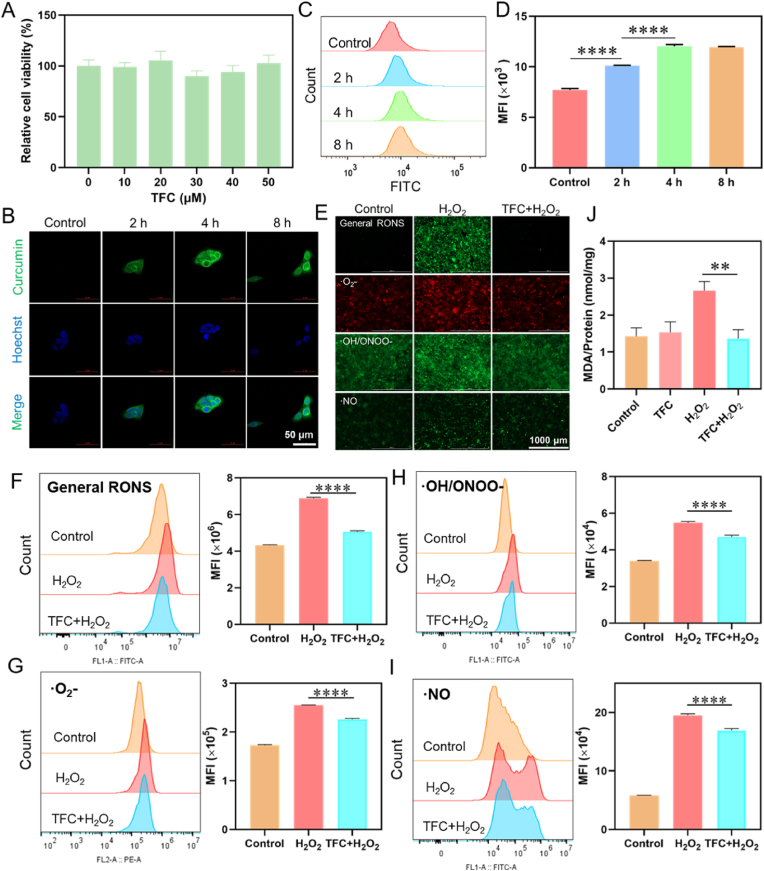
Intracellular free radical scavenging activity of TFC. (A) Cell viability of DPCs treated with different concentrations of TFC for 24 h (n = 3). (B) Confocal fluorescence images showing the cellular uptake of TFC in DPCs at different time points. (C) Flow cytometry measurement of fluorescence intensity to quantify TFC uptake by DPCs and (D) quantitative analysis (n = 3). (E) Fluorescence microscopy images showing the effect of TFC on the levels of various intracellular RONS in human dermal papilla cells after H_2_O_2_-induced oxidative stress. (F) Quantitative analysis of total RONS scavenging by TFC. Quantitative analysis of TFC-mediated scavenging of (G) superoxide anions (•O_2_^−^), (H) hydroxyl radicals (•OH)/peroxynitrite (ONOO^−^), and (I) nitric oxide (•NO) (n = 3). (J) MDA level showing the ability of TFC to alleviate oxidative stress (n = 3).

Cell viability was assessed using the MTT assay after 24 h of incubation with various concentrations of TFC (up to 50 μM, calculated as Cur). The results showed that cell viability remained above 90 %, indicating the biocompatibility of TFC at this concentration (Fig. 5A). The cellular uptake of TFC was further investigated by confocal microscopy, exploiting the spontaneous fluorescence characteristics of Cur. As the incubation time increased, the fluorescence signal inside the cells gradually became more intense, indicating a time-dependent increase in nanoparticle uptake (Fig. 5B). Flow cytometry analysis also confirmed that TFC was efficiently taken up by the cells, with the fluorescence signal plateauing after 4 h of incubation (Fig. 5C–D).

To assess the ability of TFC to scavenge intracellular RONS, we induced oxidative stress in DPCs by treating them with H_2_O_2_. The intracellular levels of general radicals, superoxide anions (•O_2_^−^), hydroxyl radicals (•OH)/peroxynitrite (ONOO^−^), and nitric oxide (•NO) were then measured using specific fluorescent probes: DCFH-DA, DHE, HPF, and DAF-FM DA. Fluorescence microscopy images revealed a significant increase in the levels of various free radicals following H_2_O_2_ treatment, indicating the induction of oxidative stress (Fig. 5E). Upon treatment with TFC, a marked reduction in the fluorescence intensity of these radicals was observed, suggesting that TFC effectively scavenges intracellular RONS (Fig. 5E and S15). Flow cytometry fluorescence quantification further confirmed that TFC significantly reduced the levels of oxidative stress markers in DPCs treated with H_2_O_2_ (Fig. 5F–I).

Oxidative stress is also known to promote lipid peroxidation, and malondialdehyde (MDA) is a well-established marker of lipid oxidation [45]. The MDA assay revealed a significant increase in MDA levels following H_2_O_2_ treatment, which was notably alleviated upon TFC treatment (Fig. 5J). These results collectively demonstrated that TFC retains its broad-spectrum free radical scavenging ability at the cellular level, providing strong evidence of its potential for mitigating oxidative stress-induced damage in DPCs.

### 3.5 Protective effect of TFC on cells under oxidative stress

Oxidative stress is a key factor in the pathogenesis of AGA, leading to the premature aging of DPCs and follicular atrophy [46]. This is often accompanied by impaired endogenous autophagy, which delays hair growth and promotes the transition from the anagen phase to the catagen phase [22]. Given the potent free radical scavenging capacity of TFC, we constructed an oxidative stress model using H_2_O_2_ to evaluate the protective effect of TFC on DPCs under oxidative stress conditions.

**Fig. 6:**
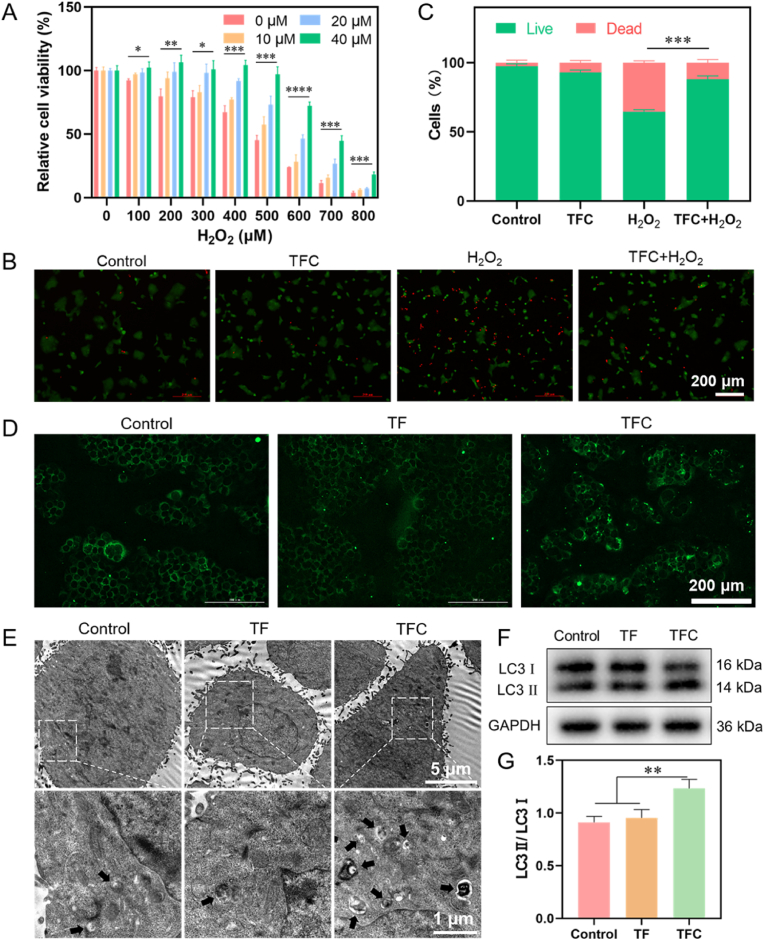
The cellular protective effect of TFC under oxidative stress. (A) The effect of TFC on cell viability following H_2_O_2_ treatment at different concentrations under oxidative stress conditions (n = 3). (B) Fluorescence microscopy images of live (green) and dead (red) cells following H_2_O_2_ treatment and TFC rescue. (C) Quantification of the live/dead cell ratio (n = 3). (D) Fluorescence microscopy images showing autophagic structures in DPCs after treatment with TFC. (E) Transmission electron microscopy images showing autophagic vesicles in DPCs after treatment with TFC. (F) Western blot analysis of LC3 expression in DPCs after treatment with TFC. (G) Semi-quantitative analysis of LC3 II expression in DPCs (n = 3). (For interpretation of the references to color in this figure legend, the reader is referred to the Web version of this article.)

Cell viability was assessed by MTT assay following treatment with various concentrations of H_2_O_2_. As the concentration of H_2_O_2_ increased, cell survival decreased in a dose-dependent manner, indicating cellular damage due to oxidative stress (Fig. 6A). TFC treatment significantly alleviated H_2_O_2_-induced cell death in a dose-dependent manner. At a concentration of 40 μM TFC, cell viability was maintained above 95 % even in the presence of 100–500 μM H_2_O_2_. To further elucidate the protective effect of TFC, DPCs were treated with 500 μM H_2_O_2_ and 40 μM TFC. Fluorescence staining of live (green) and dead (red) cells revealed a marked increase in dead cells after H_2_O_2_ treatment, with a mortality rate of 36 %. However, following TFC treatment, the number of live cells significantly increased, and the overall cell condition improved (Fig. 6B–C). These results indicated that TFC provides significant protection to DPCs under oxidative stress conditions.

Given that Cur, a component of TFC, has been demonstrated to alleviate oxidative stress and promote autophagy, we hypothesized that TFC could utilize Cur's autophagy-inducing effects to further protect cells. To test this hypothesis, we characterized autophagy levels using TF nanoparticles without Cur as a control. MDC (monodansylcadaverine), a fluorescent probe that labels acidic vacuoles, was employed to track intracellular autophagosomes. The accumulation of MDC-labeled vacuoles indicates increased autophagic activity. Fluorescence microscopy images showed a small number of bright green spots in the control and TF groups, while a significantly higher number of bright green spots were observed in the TFC group and Cur group, indicating enhanced autophagic activity induced by TFC (Fig. 6D and S16). Transmission electron microscopy further revealed that, while only a few membrane-bound autophagic vesicles were present in the control and TF groups, numerous membrane structures containing contents were observed in the TFC group, confirming an increase in autophagy (Fig. 6E).

LC3, a widely recognized marker protein for autophagy, undergoes cleavage to form LC3 I, which then associates with phosphatidylethanolamine to convert to LC3 II during autophagy [47]. Therefore, the ratio of LC3 II to LC3 I serves as an indicator of autophagic activity. Western blotting revealed a lower conversion of LC3 I to LC3 II in the control and TF groups. In contrast, the TFC group exhibited enhanced conversion, with a significantly higher expression of LC3 II (Fig. 6F–G). These findings confirmed that TFC effectively activates autophagy in DPCs, thereby exerting a protective effect against oxidative stress.

### 3.6 Preparation and characterization of TFC microneedles (MN)

**Fig. 7:**
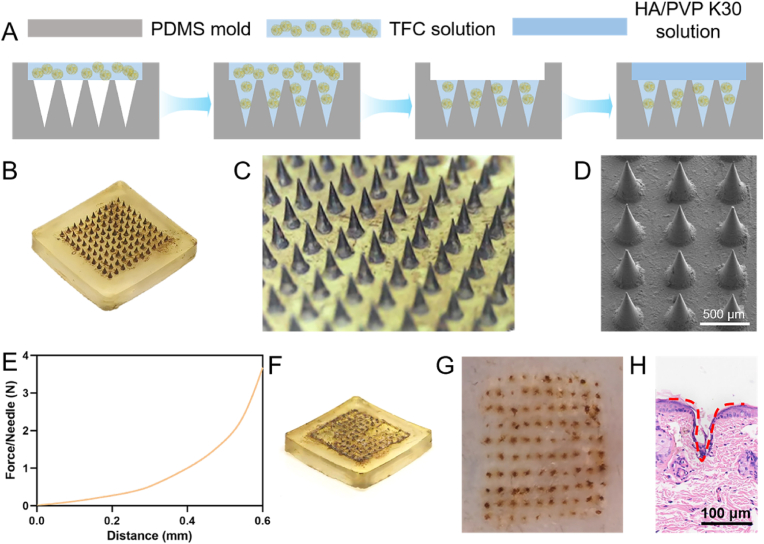
Preparation and characterization of TFC MN. (A) Schematic diagram of the preparation process for TFC MN. (B) Visual appearance of the TFC MN patch. (C) Microscopic image showing the structure of the TFC MN array. (D) Scanning electron microscopy (SEM) image of the TFC MN array. (E) Mechanical performance testing curve showing the force-displacement behavior of TFC MN. (F) TFC MN applied to ex vivo mouse skin after 5 min. (G) Image of ex vivo mouse skin treated with TFC MN, showing punctate holes corresponding to MN insertion. (H) Hematoxylin and eosin (H&E) staining of mouse skin post-TFC MN treatment.

To achieve effective transdermal delivery of TFC, we developed a microneedle (MN) system utilizing biocompatible hyaluronic acid (HA) and polyvinylpyrrolidone (PVP K30) as carriers [[48], [49], [50], [51], [52]]. TFC was incorporated into the MN through a two-step molding process, designed to overcome the skin's barrier and facilitate drug penetration (Fig. 7A). The TFC MN array consists of a 1 × 1 cm square patch, free of bubbles or cracks in the substrate layer, containing a 10 × 10 array of MN (Fig. 7B). The digital image of the MN patch reveals sharp tips, intact conical needle bodies, a neatly arranged array, and uniform distribution of the drug throughout the MN structure (Fig. 7C). Scanning electron microscopy (SEM) further confirmed the neat arrangement and conical shape of the MN. The average length of the MN was 511 μm, with a spacing of 218 μm between adjacent needles (Fig. 7D). Content analysis revealed that each MN contains 32 μg of TFC (equivalent to approximately 16.5 μg of Cur).

Biomechanical testing of TFC MN showed no fracture point during continuous compression, indicating that the MN have sufficient mechanical strength to penetrate the skin (Fig. 7E). At a displacement of 0.4 mm, each MN was subjected to a force of 1 N, which exceeds the minimum required force of 0.09 N per needle to penetrate the skin's stratum corneum. When TFC MN was applied to ex vivo mouse skin for 5 min, the MN were fully dissolved, with no remaining MN in the backing layer (Fig. 7F). Microscopic observation of the mouse skin revealed clear, punctate holes corresponding to the MN tips, with an insertion rate of 97 % (Fig. 7G). Histological analysis of hematoxylin and eosin (H&E) stained tissue sections confirmed that the MN had successfully created pores in the skin, with an insertion depth of approximately 115 μm, well beyond the thickness of the stratum corneum (∼20 μm) (Fig. 7H). In addition, the dissolution behavior of microneedles in vivo is crucial for drug delivery efficiency, which is influenced by factors such as microneedle geometry, skin elasticity, and applied force [53,54]. The in vivo dissolution results demonstrated that the TFC microneedles dissolve rapidly within 5 min (Fig. S17). These results demonstrated that TFC MN possess adequate mechanical properties and strength to effectively penetrate the skin barrier, enabling transdermal delivery of TFC.

### 3.7 Evaluation of the therapeutic effect of TFC MN in mouse AGA model

**Fig. 8:**
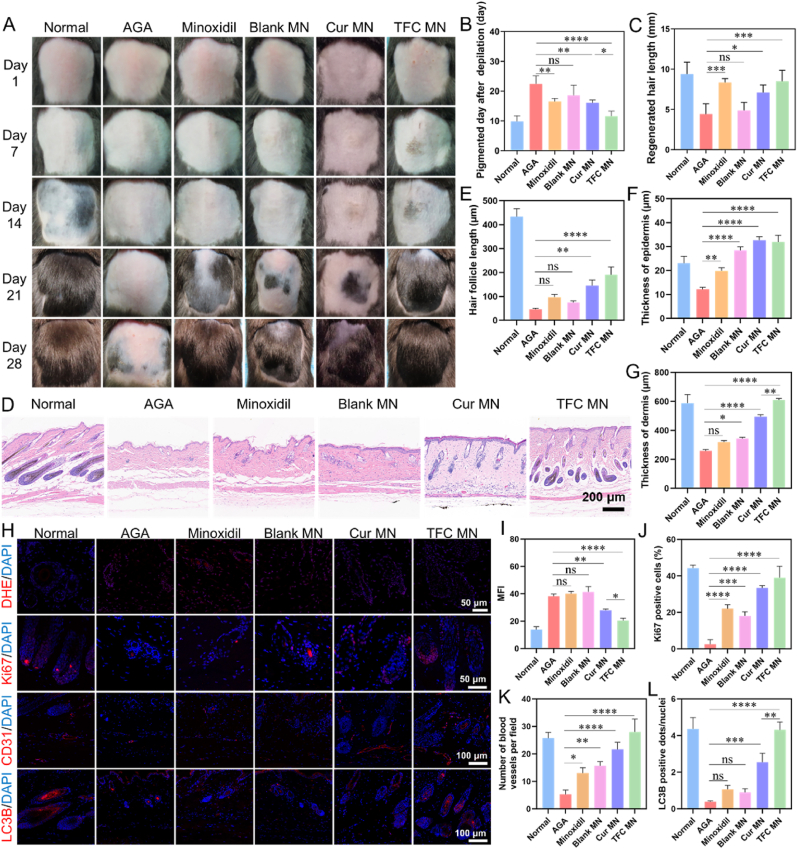
In vivo pharmacological evaluations of TFC MN. (A) Photographs showing hair regeneration in mice from each group at days 1, 7, 14, 21, and 28 post-depilation. (B) Number of days after depilation for the skin of mice in each group to turn black (n = 5). (C) Hair length measurements of newly formed hair in each group of mice on day 28 post-depilation (n = 5). (D) Hematoxylin and eosin (H&E) staining of mouse skin on day 14 post-depilation. (E) Quantitative measurement of hair follicle length in the skin of mice in each group on day 14 post-depilation (n = 3). (F) Epidermis thickness and (G) dermis thickness in the skin of mice in each group on day 14 post-depilation (n = 3). (H) Fluorescence microscopy images of reactive oxygen species (ROS), Ki67, CD31, and LC3B expression in skin tissue on day 14 post-depilation. (I) Quantification of superoxide anion levels using DHE fluorescence intensity (n = 3). (J) Quantification of Ki67 fluorescence intensity as an indicator of cell proliferation (n = 3). (K) Quantification of CD31 fluorescence intensity as a marker of angiogenesis (n = 3). (L) Quantification of LC3B fluorescence intensity as a marker of autophagy (n = 3). Blue: DAPI; Red: DHE, Ki67, CD31, LC3B. (For interpretation of the references to color in this figure legend, the reader is referred to the Web version of this article.)

To evaluate the in vivo therapeutic efficacy of TFC MN, we induced an AGA model using local testosterone application. The administration regimen is depicted in Fig. S18, and the dynamic progression of hair growth was monitored. The normal group showed pink skin following hair depilation, with hair growth commencing within one week as the skin gradually darkened (Fig. 8A). Skin color changes were used as an indicator of the hair follicle cycle. The pink color of the skin signifies the dormant phase of the hair follicles, while darkening and melanin deposition suggest the transition to the growth phase. The faster the skin darkens, the sooner hair growth is expected to occur. Thus, the time required for the skin to darken after hair depilation was used as a key indicator of hair regrowth.

The AGA model group exhibited pink skin for up to three weeks post-hair removal, with limited hair growth observed only in the fourth week, confirming the successful establishment of the model. Treatment with the positive control drug minoxidil and TFC MN both led to noticeable hair growth promotion (Fig. 8A). Interestingly, Blank MN also showed some therapeutic effect, likely due to mechanical stimulation that promotes skin tissue and microvascular regeneration, which creates a favorable environment for hair growth [55]. The time taken for the TFC MN group to achieve skin darkening was approximately 12 days, significantly earlier than the AGA group (23.5 days), Minoxidil group (16.5 days), Blank MN group (18.5 days) and Cur MN group (16 days) (Fig. 8B). On day 28, hair coverage was higher in the TFC MN group (85.48 %) compared to the AGA model group (38.28 %), Minoxidil group (73.06 %), Blank MN group (75.31 %), and Cur MN group (73.57 %) (Fig. S19). Furthermore, the TFC MN group exhibited significantly longer hair (8.49 mm) on day 28 compared to the AGA group (4.43 mm) and the Blank MN group (4.85 mm) (Fig. 8C).

Changes in hair follicle number, morphology, maturation, and skin thickness are indicative of hair growth progression. H&E staining of skin tissues on day 14 revealed that the AGA group exhibited a limited number of atrophic, immature hair follicles (Fig. 8D). While the Minoxidil, Blank MN and Cur MN groups showed the presence of some mature follicles, the majority remained in an early developmental stage. In contrast, the TFC MN group displayed a substantial number of enlarged hair bulbs and significantly elongated hair follicles, characteristic of the anagen (growth) phase (Fig. 8E). Moreover, epidermal thickness was significantly increased in all treatment groups, with the Blank MN, Cur MN and TFC MN groups showing greater epidermal thickness than the Minoxidil group, possibly due to the mechanical stimulation from microneedles that accelerates epidermal regeneration (Fig. 8F). Dermal layer thickness was also significantly increased in the TFC MN group (Fig. 8G).

To further investigate the therapeutic mechanism of TFC MN, we examined oxidative stress and cell proliferation in skin tissue. Superoxide anion levels were assessed using the DHE probe, which emits a red fluorescence when oxidised by superoxide anions and binds to DNA. The TFC MN group showed a significant reduction in superoxide anion levels compared to other groups, indicating alleviation of oxidative stress at the site of AGA (Fig. 8H–I). Ki67, a marker of cell proliferation, revealed significantly increased hair follicle cell proliferation in all treatment groups, with the TFC MN and Cur MN groups demonstrating the most substantial effect (Fig. 8H and J). CD31, a marker of endothelial cells, was used to assess angiogenesis, as increased blood flow can enhance nutrient delivery and promote hair follicle regeneration. The TFC MN and Cur MN groups exhibited the most pronounced angiogenic response, suggesting that TFC MN and Cur MN promote angiogenesis and improve the oxidative stress microenvironment in hair follicles (Fig. 8H and K). Additionally, LC3B, a key autophagy marker, was significantly upregulated in the TFC MN and Cur MN group, confirming the activation of autophagy in hair follicle cells, which likely contributed to the transition of AGA hair follicles from the telogen phase to the anagen phase (Fig. 8H and L). Further, immunofluorescence results confirmed the mechanism of TFC in alleviating oxidative stress and activating autophagy, where the expressions of p-c-Jun, p-Akt, p-mTOR were decreased in the TFC MN group compared with the AGA group (Fig. S20). These results collectively demonstrated that TFC MN promotes hair regeneration in AGA mice by reducing oxidative stress, activating autophagy in hair follicle cells, and enhancing cell proliferation and angiogenesis.

Finally, the in vivo safety of TFC MN was assessed. Throughout the experimental period, no significant changes were observed in the body weight of the mice, and no major organ damage was detected (Fig. S21 and S22). Furthermore, no iron accumulation was found in major tissues or organs following TFC MN treatment (Fig. S23), indicating that TFC MN is safe for in vivo use.

## 4 Conclusion

In conclusion, this study provides compelling evidence that TFC, a Cur-based nanoparticle formulation, effectively addresses the key pathological mechanisms underlying AGA. By combining the antioxidant and autophagy-promoting properties of Cur, TFC not only alleviates oxidative stress in the hair follicle microenvironment but also stimulates hair follicle regeneration through autophagic activation. The microneedle-based delivery system (TFC MN) offers a promising transdermal strategy to enhance the therapeutic potential of Cur, overcoming its limitations in bioavailability. In vivo experiments demonstrated significant improvements in hair regeneration, follicle proliferation, and angiogenesis, with minimal side effects, highlighting the potential of TFC as an innovative and safe therapeutic approach for AGA. These findings open new avenues for the clinical treatment of hair loss disorders, underscoring the therapeutic value of nanotechnology in drug delivery systems for dermatological applications.

## CRediT authorship contribution statement

**Yuanzheng Chen:** Writing – original draft, Methodology, Investigation, Formal analysis, Conceptualization. **Qubo Zhu:** Validation, Methodology, Investigation. **Yanbin Zhou:** Methodology, Investigation. **Wenhu Zhou:** Writing – review & editing, Supervision, Funding acquisition, Conceptualization. **Yan Chen:** Supervision, Conceptualization.

## Declaration of competing interest

The authors declare that they have no known competing financial interests or personal relationships that could have appeared to influence the work reported in this paper.

## Data Availability

Data will be made available on request.
