# Supplementary material for: Multifunctional nanomedicine targeting the 'seed-and-soil' of hair follicles via simultaneous alleviation of oxidative stress and activation of autophagy for androgenetic alopecia therapy
Source: Mater Today Bio. 2025 Jul 29;34:102145. doi: 10.1016/j.mtbio.2025.102145 (PMC12332918; doi:10.1016/j.mtbio.2025.102145)
Supplement: Multimedia component 1 [file mmc1.docx]

**Supporting Information**

**Multifunctional Nanomedicine Targeting the 'Seed-and-Soil' of Hair Follicles via Simultaneous Alleviation of Oxidative Stress and Activation of Autophagy for Androgenetic Alopecia Therapy**

Yuanzheng Chen^a^, Qubo Zhu^a^, Yanbin Zhou^a^, Wenhu Zhou*^a, c^, Yan Chen*^b^

^a^ Xiangya School of Pharmaceutical Sciences, Central South University, Changsha, Hunan, 410013, China

^b^ Quzhou Hospital Affiliated to Wenzhou Medical University (Quzhou People's Hospital), Quzhou, 324000, China

^c^ Hunan Key Laboratory of The Research and Development of Novel Pharmaceutical Preparations, School of Pharmaceutical Science, Changsha Medical University, Changsha, 410219, China

*Corresponding authors：

E-mail: [zhouwenhuyaoji@163.com](mailto:zhouwenhuyaoji@163.com)

qzchenyan@wmu.edu.cn;


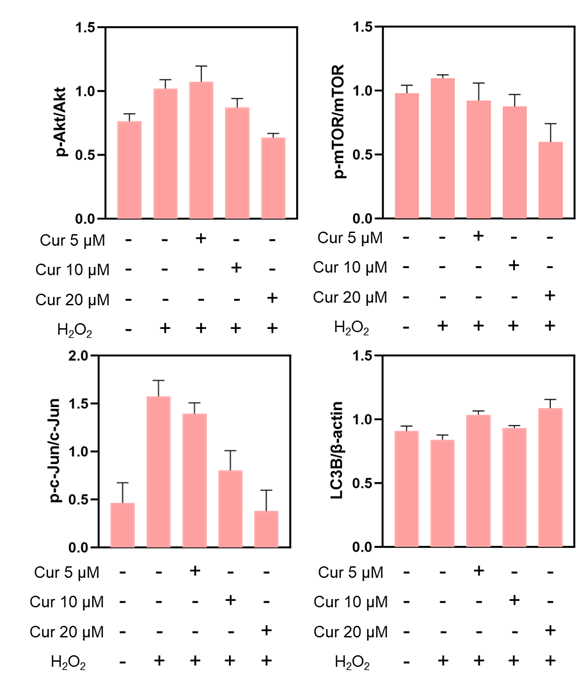


Fig. S1. Expression levels of p-Akt, p-mTOR, p-c-Jun and LC3B in DPCs of different treatment groups (n=3).


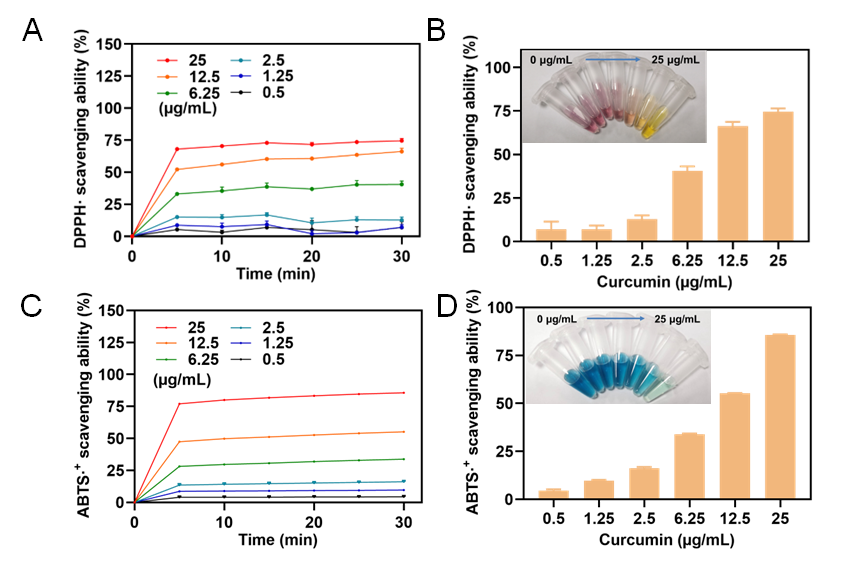


Fig. S2. (A) DPPH· scavenging by different concentrations of Cur over time (n=3). (B) Scavenging of DPPH· by different concentrations of Cur (n=3). (C) Scavenging of ABTS·^+^ by different concentrations of Cur over time (n=3). (D) Scavenging rate of ABTS·^+^ by different concentrations of Cur (n=3).


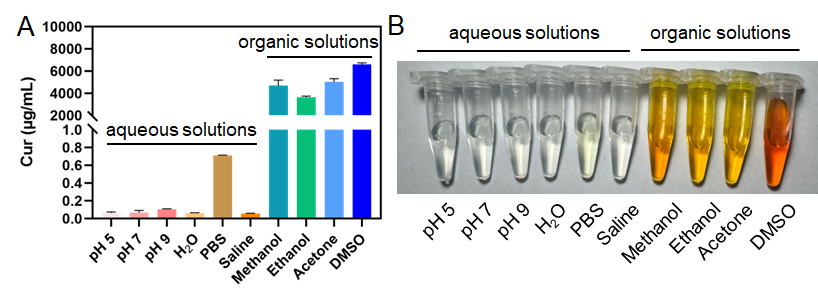


Fig. S3. (A) The concentration of curcumin dissolved in aqueous and organic solutions (n=3) and (B) color of various solutions.


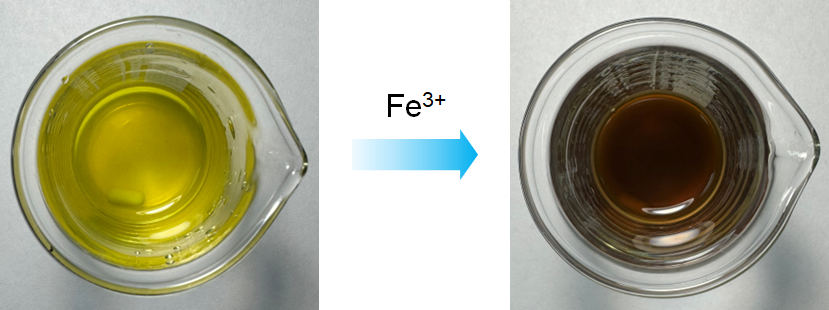


Fig. S4. Color change of the solution before and after addition of iron ions.


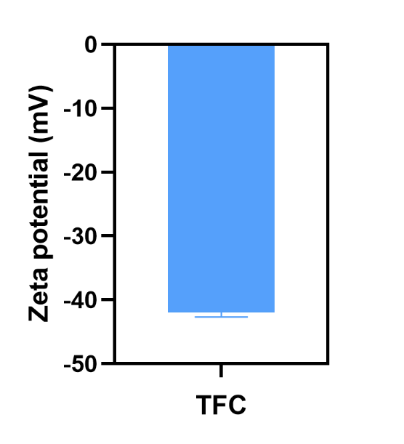


Fig. S5. Zeta potential of TFC (n=3).


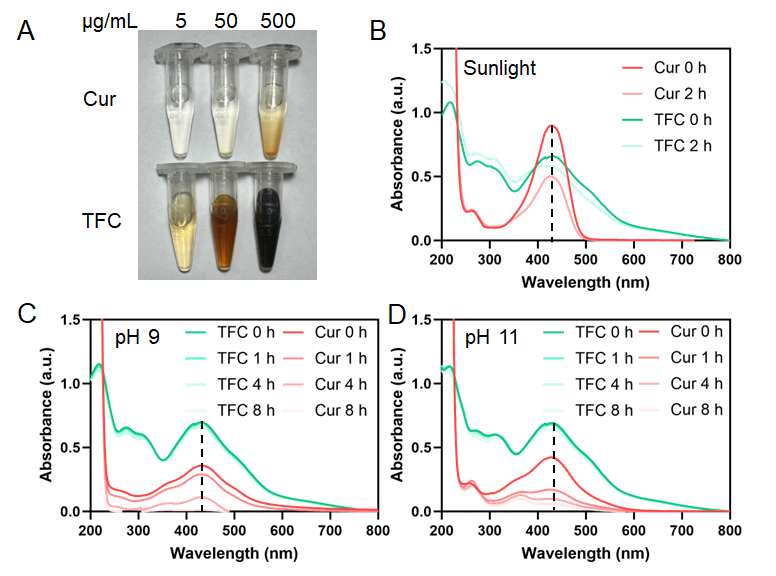


Fig. S6. (A) Dispersion states of different concentrations of Cur and TFC (measured as Cur) in ultrapure water. Stability of Cur and TFC at (B) sunlight, (C) pH 9 and (D) pH 11.


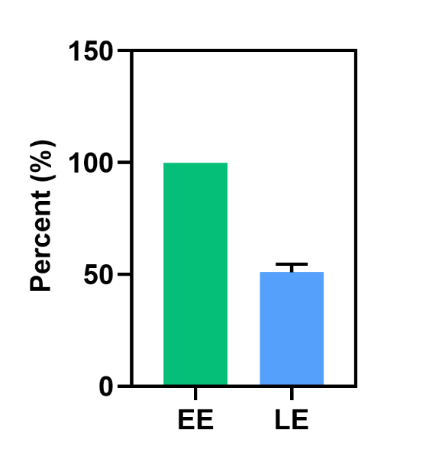


Fig. S7. Encapsulation efficiency and drug loading efficiency of Cur in TFC (n=3).


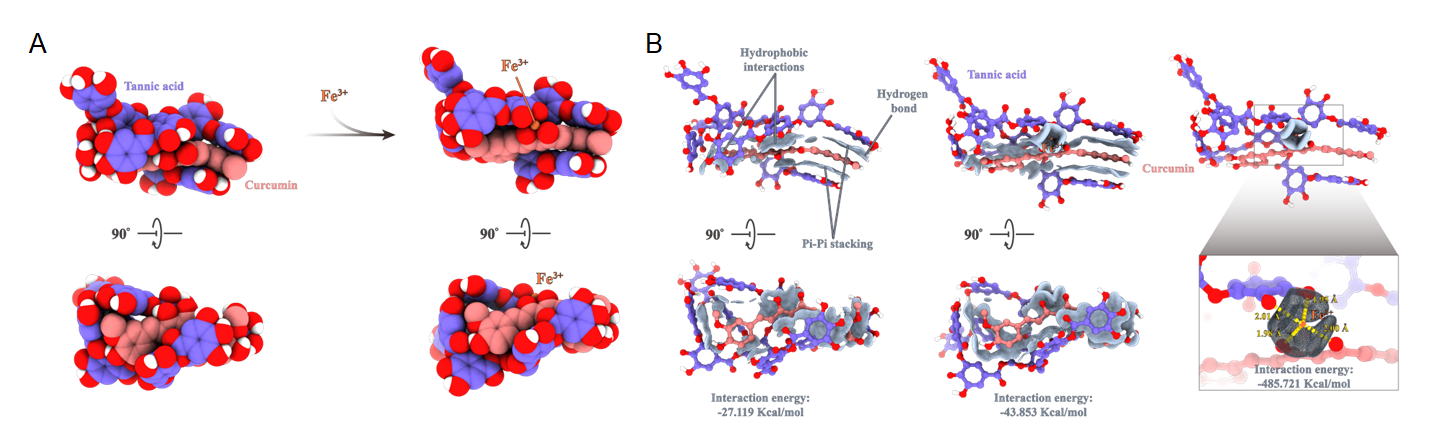


Fig. S8. (A) Conformational analysis of the interaction between tannic acid, curcumin, and Fe^3+^. (B) Interaction pattern and energy analysis of tannic acid, curcumin, and Fe^3+^.


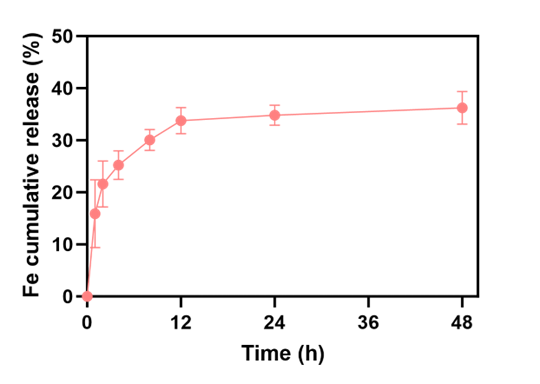


Fig. S9. Cumulative release of Fe in Tris-HCl (pH 7.4, containing 1% mouse skin homogenate) (n=3).


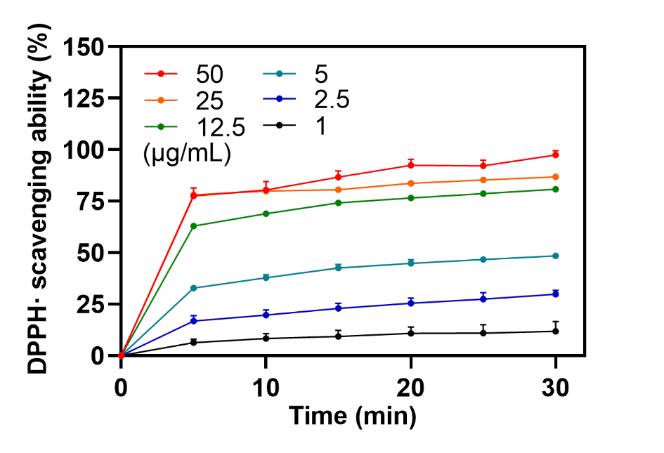


Fig. S10. DPPH· scavenging over time by different concentrations of TFC (n=3).


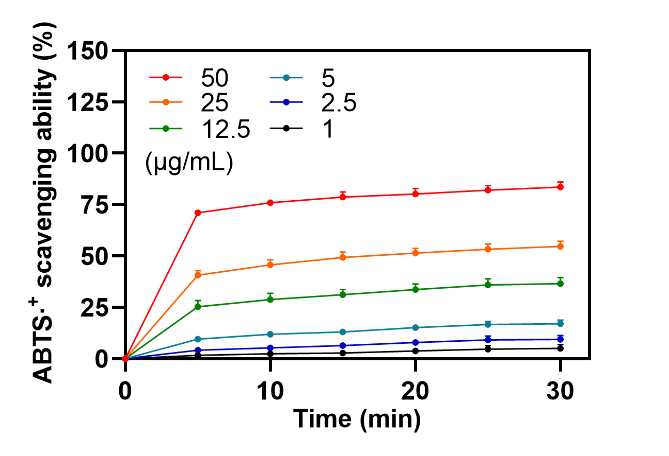


Fig. S11. ABTS·^+^ scavenging over time with different concentrations of TFC (n=3).


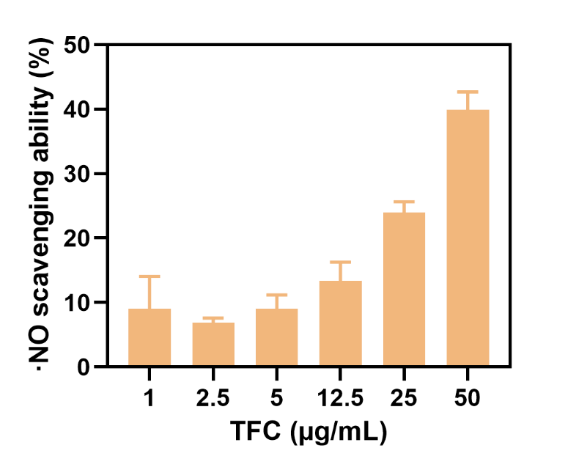


Fig. S12. Scavenging of ·NO by different concentrations of TFC (n=3).


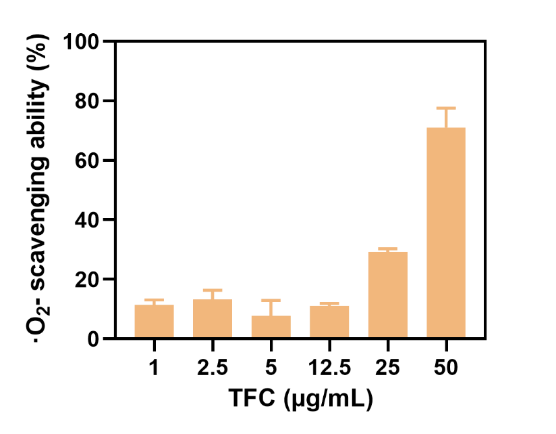


Fig. S13. Scavenging of ·O_2_- by different concentrations of TFC (n=3).


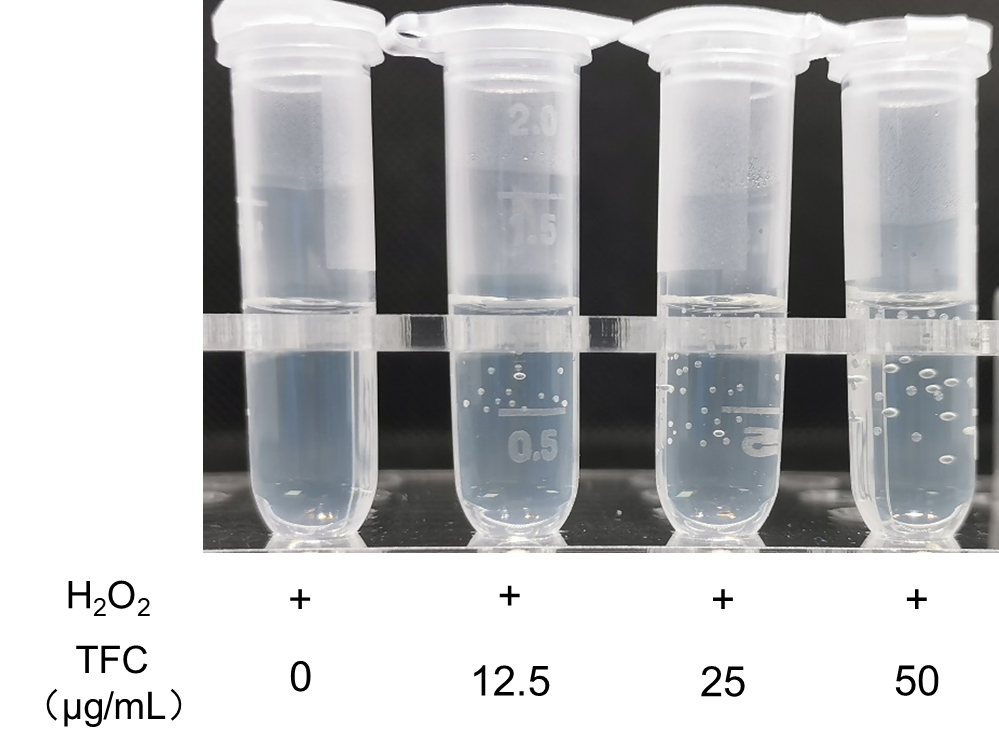


Fig. S14. Oxygen production from decomposition of H_2_O_2_ by different concentrations of TFC.


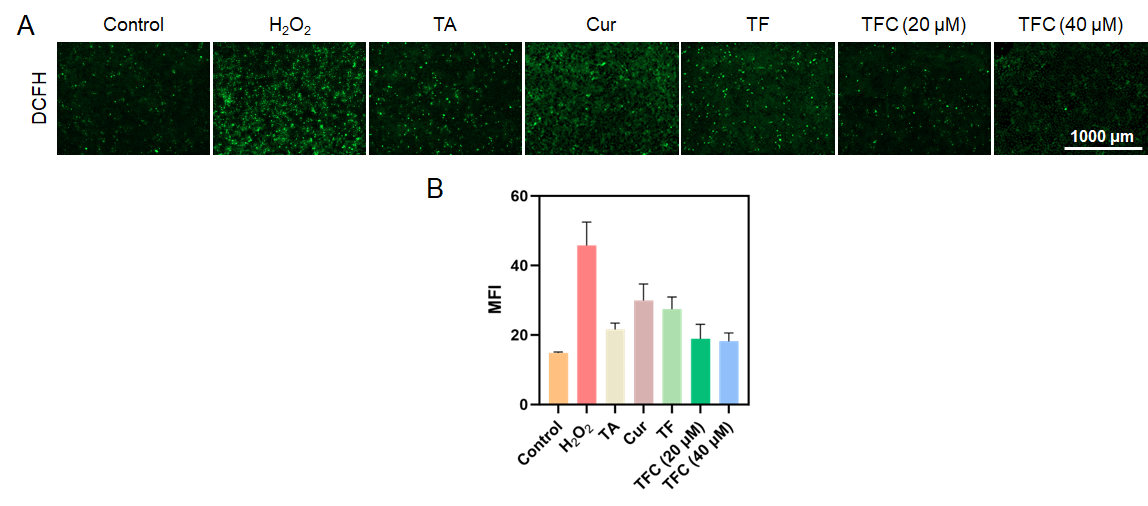


Fig. S15. (A) Fluorescence images of intracellular ROS levels and (B) mean fluorescence intensity in different treatment groups (n=3).


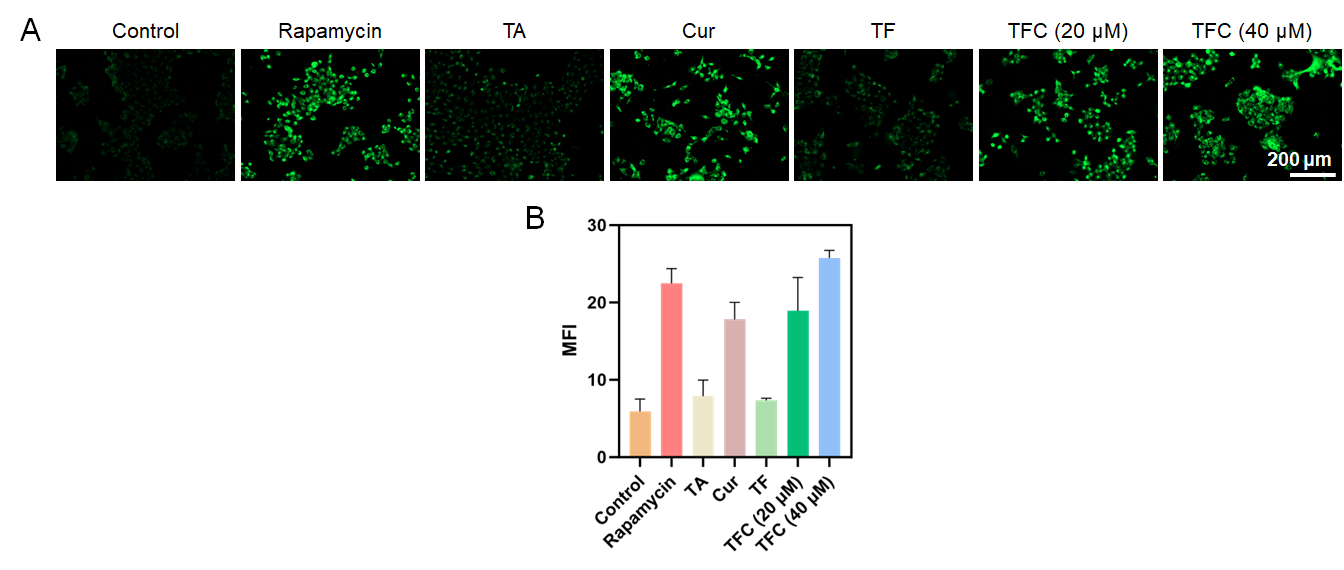


Fig. S16. (A) Fluorescence images of intracellular autophagy levels and (B) mean fluorescence intensity in different treatment groups (n=3).


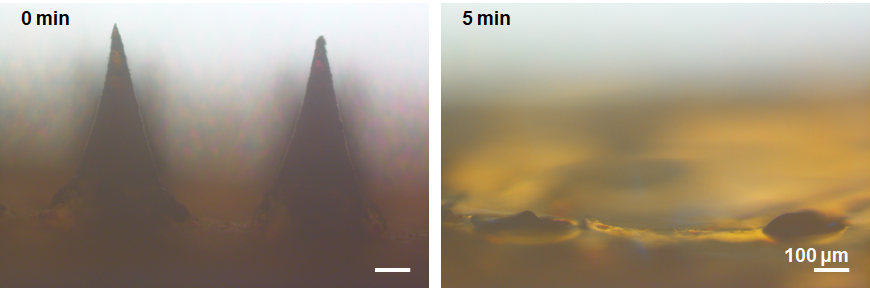


Fig. S17. In vivo dissolution behavior of TFC MN.


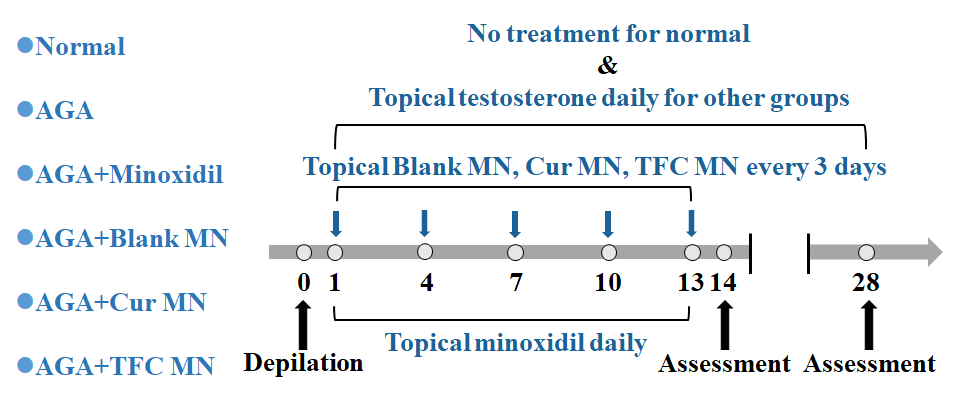


Fig. S18. Scheme of AGA modeling and administration of drugs in each group.


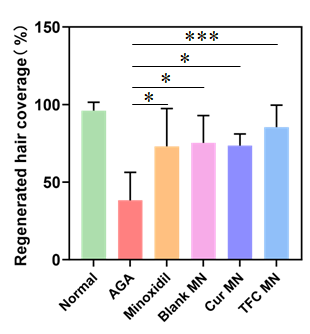


Fig. S19. Hair coverage of mice in different treatment groups on day 28 after hair depilation (n=5).


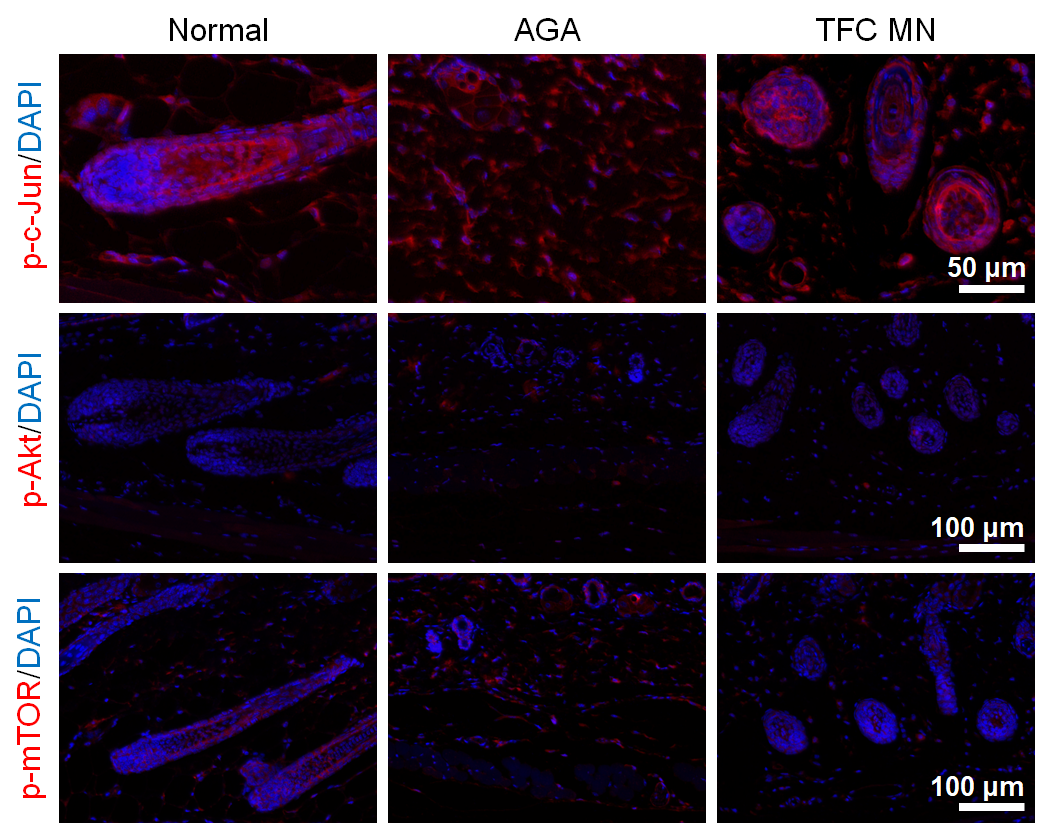


Fig. S20. Fluorescence microscopy images of p-c-Jun, p-Akt and p-mTOR expression in skin tissue on day 14 post-depilation.


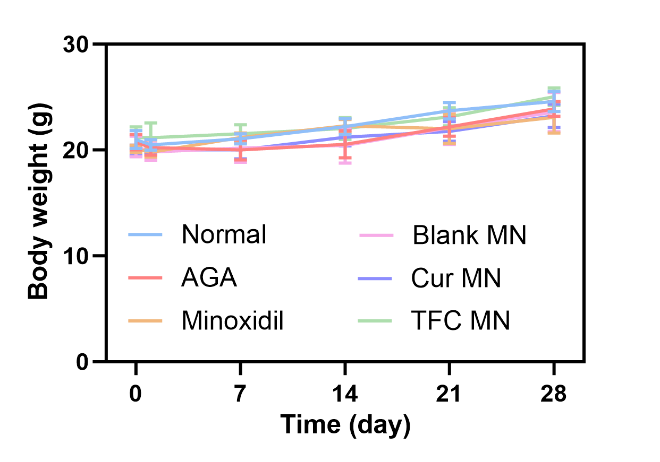


Fig. S21. Changes in body weight of mice in each group (n=5).


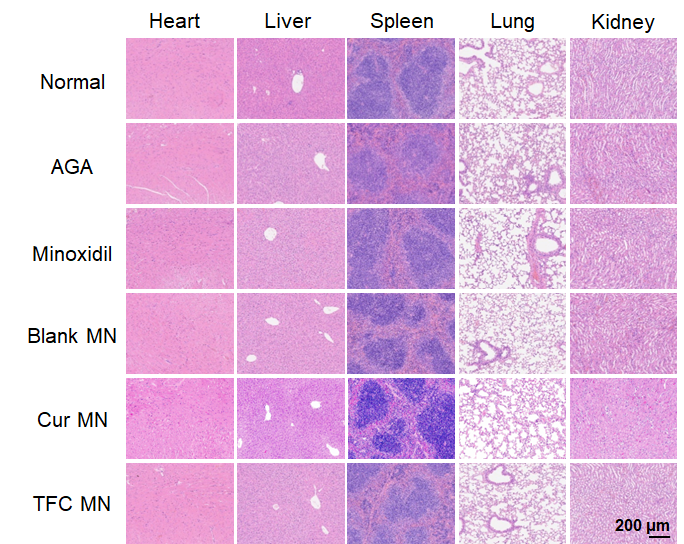


Fig. S22. H&E sections of heart, liver, spleen, lungs, and kidneys from various groups of mice.


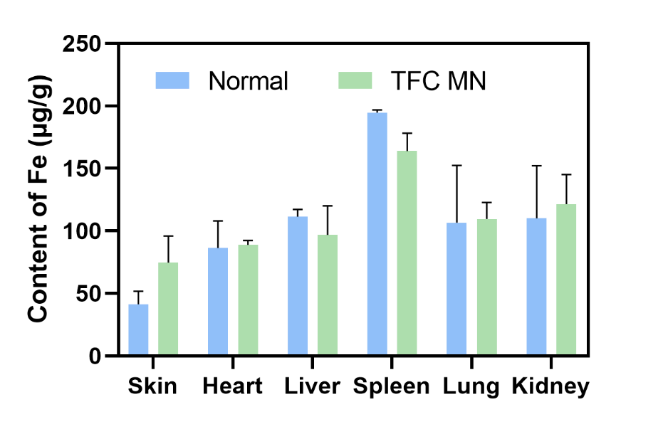


Fig. S23. Iron content in the skin, heart, liver, spleen, lung, and kidney of mice in the normal group and TFC MN group (n=3).
